# Insight on physicochemical properties governing peptide MS1 response in HPLC-ESI-MS/MS: A deep learning approach

**DOI:** 10.1016/j.csbj.2023.07.027

**Published:** 2023-07-22

**Authors:** Naim Abdul-Khalek, Reinhard Wimmer, Michael Toft Overgaard, Simon Gregersen Echers

**Affiliations:** Department of Chemistry and Bioscience, Aalborg University, Aalborg 9220, Denmark

**Keywords:** Quantitative proteomics, ESI-MS, Deep learning, Attention mechanism, MS1 response prediction, Absolute quantification

## Abstract

Accurate and absolute quantification of peptides in complex mixtures using quantitative mass spectrometry (MS)-based methods requires foreground knowledge and isotopically labeled standards, thereby increasing analytical expenses, time consumption, and labor, thus limiting the number of peptides that can be accurately quantified. This originates from differential ionization efficiency between peptides and thus, understanding the physicochemical properties that influence the ionization and response in MS analysis is essential for developing less restrictive label-free quantitative methods. Here, we used equimolar peptide pool repository data to develop a deep learning model capable of identifying amino acids influencing the MS1 response. By using an encoder-decoder with an attention mechanism and correlating attention weights with amino acid physicochemical properties, we obtain insight on properties governing the peptide-level MS1 response within the datasets. While the problem cannot be described by one single set of amino acids and properties, distinct patterns were reproducibly obtained. Properties are grouped in three main categories related to peptide hydrophobicity, charge, and structural propensities. Moreover, our model can predict MS1 intensity output under defined conditions based solely on peptide sequence input. Using a refined training dataset, the model predicted log-transformed peptide MS1 intensities with an average error of 9.7 ± 0.5% based on 5-fold cross validation, and outperformed random forest and ridge regression models on both log-transformed and real scale data. This work demonstrates how deep learning can facilitate identification of physicochemical properties influencing peptide MS1 responses, but also illustrates how sequence-based response prediction and label-free peptide-level quantification may impact future workflows within quantitative proteomics.

## Highlights

• Peptide MS1 response was investigated using a deep learning model.
• Data from > 4000,000 equimolar peptides was extracted from repository data.
• The model attention mechanism identified key physicochemical properties.
• Hydrophobicity, charge, and structural properties affect peptide MS1 responses.
• Log-transformed intensity was predicted with 11% error based only on sequence input.

## Graphical Abstract

**Figure:**
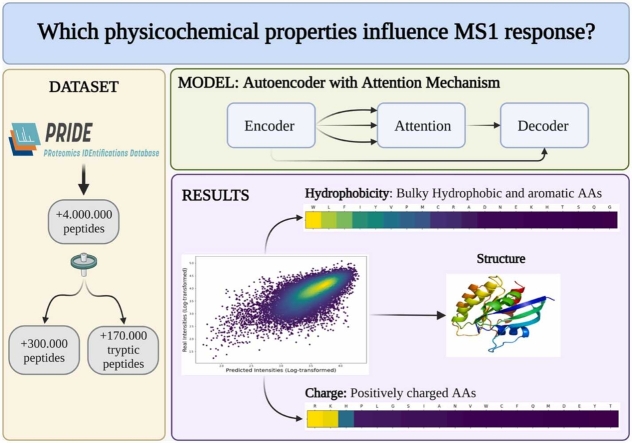

## 1 Introduction

Mass spectrometry (MS) is a very powerful method for the identification and quantification of a wide range of biomolecules present in complex mixtures and has become a cornerstone in the studies of proteins and peptides [1], [2], [3], [4], [5], [6]. In proteomics and peptidomics analyses, MS is often used in combination with other technologies, particularly chromatography-based methods such as high performance liquid chromatography (HPLC). Initially, analytes are ionized, usually by soft ionization methods such as electrospray ionization (ESI), and then discriminated by the mass analyzer based on the mass-to-charge ratio (*m/z*) [7]. However, limitations for absolute quantification remain due to variability in the ionization efficiency between different biomolecules, directly implying that MS is not inherently quantitative [8], [9], [10]. Nevertheless, by development of data normalization strategies, it is possible to develop methods for label-free, relative quantification of proteins using MS [11], [12]. In contrast, absolute quantification by MS requires prior knowledge about the compound(s) to be quantified to develop targeted approaches. Moreover, a standard series or the addition of isotopically labelled reference standards in known concentrations is required to quantify each compound. Thus, absolute quantification methods introduce restraints and limitations to the number of compounds that can be quantified, but also introduce higher analytical complexity and cost for MS analysis [13], [14], [15], [16]. While efforts have been made towards absolute, label-free quantification on the protein-level [17], [18], these approaches rely on fundamental assumptions regarding the sample composition and thus limits the applicable range to protein-level quantification for samples of certain origin. Ultimately, there is a need to develop new and universally applicable methods for absolute MS-based quantification on the peptide-level without a priori knowledge of the mixture composition. Raw MS1 intensities have been used as a rough pseudo-estimate of peptide abundance [19], [20], [21], using the same basis of assumptions employed in quantitative summary-based methods for protein-level quantification [22]. Nevertheless, such an approach does not alleviate the large uncertainties associated with differential ionization efficiency (occasionally referred to as unequal measurability of peptides [23]) in a satisfactory manner. To address this challenge, artificial intelligence (AI) is making headway for bringing novel solutions to the field of MS-based proteomics [24].

AI is a branch of computer science that focuses on developing systems able to perform tasks which require human like capabilities [25]. More specifically, machine learning (ML) and deep learning (DL) are data-centric approaches to develop models to perform specifics tasks. In the field of protein science, ML and DL have facilitated substantial advances for the prediction of e.g., protein structure, protein function, and protein-protein interactions [26], [27], [28], but is also becoming increasingly popular within MS-based proteomics. For example, ML- and DL-models have been developed predict peptide retention time, MS/MS fragmentation spectra, and post-translational modifications [24], [29], [30]. Currently, there are no computational methods to perform absolute peptide quantification based on MS response, since the ionization efficiency, and thus MS response, varies widely between individual peptides [24]. In addition to the physicochemical properties of the analytes (e.g. peptides), the experimental setup is bound to influence the results obtained [31].

Within ML and DL, recurrent neural networks (RNNs) are of particular interest within MS-based proteomics. These network architectures consider not only the current element of input but also previous ones, making RNNs ideal for sequential and time-series data [25]. Sequence-to-sequence RNNs (Seq2Seq) is an arrangement of RNNs that has shown great success in problems like language translation [32]. A Seq2Seq consists of two components: an encoder and a decoder. The encoder initially receives and transforms the inputs to generate the context vector. The transformation performed by the encoder can serve different purposes such as feature extraction and/or dimension reduction. Then, the decoder uses the context vector to generate the output. In Seq2Seq RNNs, the encoder is responsible for compressing the input data into a fixed-dimensional vector that the decoder uses to sequentially generate the output. However, compressing large quantities of information into a single vector can be a computationally heavy task. This could be improved by an attention mechanism., which enables the decoder to access all encoder outputs and focus only on the most relevant elements when predicting each element of the output sequence [33]. An encoder-decoder with an attention mechanism has previously been applied on peptide-level MS data for prediction of peptide fragmentation spectra and retention times [34], and may also be suitable to predict the peptide precursor intensity response (MS1) for application in peptide quantification. Although not a sequence-to-sequence problem but more a sequence-to-scalar problem, the attention mechanism can focus on specific elements within the sequence and thus provide deeper insight into how peptide composition affects the MS response.

In recent years, a number of tools have been developed that exploit ML and DL for prediction of proteotypic peptides, such as AP3 [35], PeptideRanger [36], CONSeQuence [37], [38], and d::pPop [39], [40]. Proteotypic peptides are peptides that are well suited for MS analysis as they are released through common sample preparation (i.e., tryptic digest) and are likely to be ionizable and detectable [41]. This makes such peptides optimal choices for e.g., relative quantification between samples in targeted/data-independent analysis or as isotopically labeled surrogates for absolute quantification [42], [43]. These tools were trained, in part, using computed physicochemical properties based on amino acid sequences, which allow them to predict peptide detectability. While the models find hidden patterns in data related to e.g. certain physicochemical properties, they do not provide any direct insight into these patterns nor provide explicit quantitative information. Repurposing of repository data to build sufficiently large datasets suitable for DL may represent a key step for further development towards label-free absolute quantification on the peptide-level [44]. Compiling repositories as well as systematic metadata annotation, data extraction, and preprocessing has therefore also become increasingly important and popular [45].

In this study, we investigate the current largest repository collection of equimolar peptide MS data [34], [46], [47]. a DL model (encoder-decoder with an attention mechanism) that uses amino acid (AA) composition only to predict MS1 intensity and provide insight on the physicochemical properties that govern peptide MS1 response in HPLC-ESI-MS/MS analysis. Thus, instead of using computed physicochemical properties, as in previous studies, our model will identify the relevancy of each AA through its attention weight. The attention weights can then be correlated with their correspondent physicochemical properties using the AAindex1 (Amino Acid Index) database [48]. This database is a public collection of 566 indices that describe the physicochemical or structural properties and propensities of individual AAs. Each index consists of a set of 20 values that correspond to a specific property of each AA. The results obtained in this study provide a better fundamental understanding of the behavior of peptides within the mass spectrometer. Moreover, we developed a model to predict peptide MS1 response as a function of AA composition. The presented work is of great relevance for the development of more advanced models to predict e.g., peptide detectability and to facilitate advances in label-free, absolute peptide quantification.

## 2 Materials and methods

### 2.1 Data

The experimental data used in this study was collected from the PRIDE repository with the identifiers PXD004732 [46], PXD010595 [34], and PXD021013 [47]. The datasets were originally obtained by analyzing pools of approximately 1000 synthetic peptides with equimolar concentrations. The data originates from development of Prosit [34] and ProteomeTools [46], [47], with the intention of boosting peptide identification rates and improving sensitivity in tandem MS by application of DL for predicting fragmentation spectra. Due to the equimolar nature of the analyzed pools, the datasets serve as an excellent basis for investigating sequence-dependent responses. RAW data was analyzed using either specific, semi-specific, or unspecific *in silico* digestion settings in MaxQuant and with Trypsin, LysN, or AspN as specified protease. In all studies, peptide pools were subjected to liquid chromatography using a Dionex 3000 HPLC system (Thermo Fisher Scientific) coupled inline with an Orbitrap Fusion Lumos mass spectrometer (Thermo Fisher Scientific) [34], [46], [47]. This ensured experimental comparability between studies and was a prerequisite for inclusion in the database compiled for this study. From the peptide-level MaxQuant output files (peptides.txt, summary.txt) and sample and data relationship file (SDRF), several data features were extracted and processed using a custom Python (v.3.8.8) script. Each pool analyzed had a corresponding zip file containing the peptide.txt and summary.txt files. The final results of each analysis were extracted from the peptide.txt file (sequence of identified peptides, MS1 intensities, PEP scores, etc.) while the specified enzyme and enzyme mode settings were extracted from the summary.txt files. The SDRF files contains information relating each pool zip file with its specific experimental setups. A unique CSV file was generated for each pool unifying the information in the previously mentioned files, and subsequently merged into a single CSV file comprising all the information of the repositories PXD004732, PXD010595 and PXD021013. Artificial datasets were also generated to build proof-of-concept models, representing simple linear datasets and datasets with larger variability between contributions (see supplementary material for a detailed description).

### 2.2 Data filtering and pre-processing

To build the best possible model, the data (4016,044 identified peptides) was initially filtered with the intention of reducing noise, thereby improving data quality for the training and testing process of the models. The artificial data did not require filtering. The data was initially filtered using quality-based criteria:

- All peptide sequences with a PEP score equal to or higher than 0.01were removed (587,374 peptides).
- Reverse sequences were excluded (414 peptides).
- Peptides determined as potential contaminants were not considered (22,257 peptides).
- Peptides with intensity measurement equal to zero were discarded (40,798 peptides).

Following initial filtering, the dataset was further processed and filtered using replication- and variation-based criteria:

• Peptide replicates across different pools were merged (2316,063 peptides). For each peptide, the median intensity was used for the analysis.
• Peptides with intensity values comprising a coefficient of variation (CV) higher than 0.3 (standard deviation divided by mean) were excluded (728,397 peptides).

The final dataset consisted of 320,741 unique peptide entries with replicate values.

#### 2.2.1 Further data segmentation

To further improve the model’s performance, we segmented the data according to specified MaxQuant settings, restricting focus to tryptic peptides with repeated measurements:

• Peptides that were not searched with “Specific Enzyme” mode were removed (1598,623 peptides).
• Non-tryptic peptides were discarded (184,918 peptides)
• Replicate peptide measurements were merged (1177,976 peptides). For each peptide, the median intensity was used for the analysis.
• Peptides with intensity values with a coefficient of variation higher than 0.3 were dismissed (224,462 peptides).

The final number of peptides in the tryptic dataset was 179,222.

#### 2.2.2 Transformation, scaling, and splitting

Following filtering, peptide intensity values (which are continuous values) were log-transformed (natural logarithm) because intensity values show an exponential behavior over a large dynamic range. The log transformation generates a distribution closer to normal. The intensity values were scaled between a specific range of values, using the MinMaxScaler function from Scikit-learn library [49], which was optimized (the same was done to artificial data) by trying different ranges to improve model performance. Log-transformation and scaling of intensity data is commonly used to obtain a more normal distribution in MS-based proteomics data [50], [51], [52], [53], [54]. To validate reproducibility of model performance, a 5-fold cross-validation was performed, where the dataset was split into 5 groups of equal size, then each unique group was used as test set while the remaining 4 groups were used as training sets. Thus, 80% of the data was used for training, and 20% for testing. From the training data, 20% was randomly subset and used as a validation dataset to control overfitting. The test dataset was used to evaluate the generalization capacity, to give an unbiased evaluation of the models, and to obtain the results.

### 2.3 Model architecture

The model architecture is an RNN encoder-decoder with attention mechanism [55], [56]. The function and purpose of the different elements of the architecture are presented below and a description of the complete end-to-end pipeline is available in the supplementary information.

#### 2.3.1 Recurrent neural networks (RNNs)

RNNs process input data by iterating through the elements of the input, while keeping a memory or state from previous elements of the input [25]. RNNs take an input sequence X = {*x*_*1*_, *x*_*2*_, *x*_*3*_, …, *x*_*T*_} one element at a time to compute an output sequence Y= {*y*_*1*_, *y*_*2*_, *y*_*3*_, …, *y*_*T*_}. The output y_t_ at step t (which can represent time-resolved data or other sequential inputs) is defined as:

$$y_{t}=f(x_{t},h_{t-1})$$

where *h*_*t-1*_ is the previous hidden state and *f* is a non-linear function.

The three most common type of RNNs are the simple RNNs, the long short-term memory neural network (LSTM), and the gated recurrent unit neural network (GRU) [33]. The simple RNNs iterates over elements in a sequence, considering the previous state and current input to generate the current output and then uses the current output as the state of the next element in the sequence. Simple RNNs have problems keeping long-term dependencies when working with long sequences due to the vanishing gradient problem [57], which is why LSTM and later GRU were developed. LSTM and GRU layers can keep information for longer, thereby improving the predictive capabilities. While displaying comparable performances, GRU layers are simpler and easier to train [33], [55].

A GRU consists of cells that contain gates, which are responsible of determining which information is relevant and should be retained and which is irrelevant and can be forgotten. GRU layers have two gates (update gate z, reset gate r), a candidate hidden state h’, and a hidden state at the current time step h. The update gate determines how much of past information is relevant now. The reset gate, in contrast, decides how much of the past information to forget. The hidden state at the current step is a linear interpolation between the previous hidden state and the current candidate hidden state [55].

#### 2.3.2 Sequence to sequence RNNs

Seq2Seq RNNs consist of an encoder RNN and a decoder RNN [32], [58]. When given an input sequence X = {*x*_*1*_, *x*_*2*_, *x*_*3*_, …, *x*_*T*_} the Seq2Seq maps the prediction to an output sequence Y= {*y*_*1*_, *y*_*2*_, *y*_*3*_, …, *y*_*N*_} with potentially different lengths. The input sequence X is passed to the encoder RNN one step at a time, in order to generate a context vector c. The context vector is a fixed-dimensional vector that encodes the input sequence. The context vector is passed to the decoder RNN, which unfolds it, one step at a time, to generate the output sequence Y.

#### 2.3.3 Attention mechanism

An encoder-decoder arrangement, such as Seq2Seq, has certain limitations due the fact that the encoder needs to compress all the input data into the context vector, which can lead to loss of information, which may ultimately affect the performance. Thus, the attention mechanism was developed which allows the model to focus on the most relevant elements of the input sequence based on the determined attention weights [59]. Particularly, Bahdanau attention [60] calculates a linear combination of the encoder and decoder states. The attention weights represent the degree of attention that should be given to each input element at a particular decoding stage. At each stage, the context vector is generated using all the hidden states from the encoder and the previous hidden state from the decoder. The context vector c_*i*_ is calculated as the weighted sum of the encoder hidden states:

$$c_{i}=\sum_{j=1}^{T}\alpha_{\mathrm{ij}}h_{j}$$

where the attention weight *α*_*ij*_ of each hidden state *h*_*j*_ is calculated as:

$$\alpha_{\mathrm{ij}}=\frac{\exp\left(e_{\mathrm{ij}}\right)}{\sum_{k=1}^{T}\exp\left(e_{\mathrm{ik}}\right)}$$

and the attention scores *e*_*ij*_ is defined as:

$$e_{\mathrm{ij}}=a\left(s_{i-1},h_{j}\right)$$

where *a* is a function that generates the attention scores (*e*_*ij*_) that assign how well *s*_*i-1*_ and *h*_*j*_ match. *s*_*i-1*_ is the decoder hidden estate (before generation the output at *i*) and *h*_*j*_ in the encoder hidden state at *j*.

**Fig. 1:**
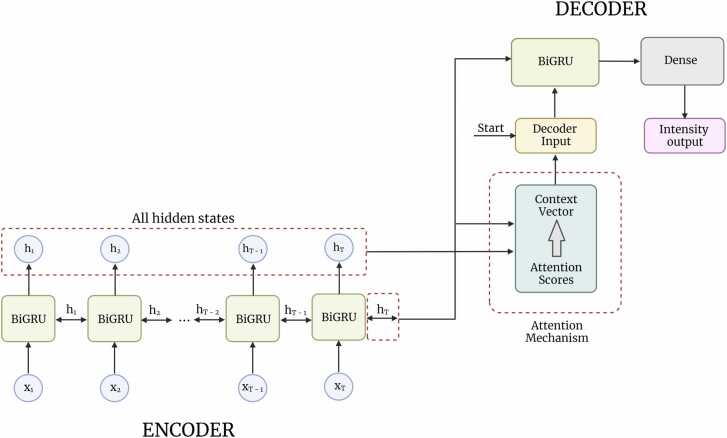
General scheme of the architecture used in this study: An encoder-decoder with attention mechanism. The encoder consists of one BiGRU layer, which takes the inputs (x_i_,…,x_T_) and generates the encoder hidden states (h_i_,…,h_T_). All the hidden states from the encoder and the last hidden state from the decoder is used by the attention mechanism to compute the context vector, which together with the start character for the decoder are used as the decoder input. The decoder consists of one BiGRU layer and a dense layer. The first and only initial state of the decoder is the last hidden state of the encoder. The decoder only performs one iteration since the decoder output is a scalar and not a sequence.

The model architecture used in this study is an encoder-decoder based on a bi-directional recurrent neural network layer with Gated Recurrent Units (BiGRU) and with an attention mechanism (Fig. 1). The encoder consists in one BiGRU layer. All hidden states of the encoder and the last hidden state of the decoder are used to compute the attention weights and subsequently the context vector. The context vector is then concatenated with the one-hot-encoded start element for the decoder to generate the decoder input. The decoder also has one BiGRU in addition to a dense layer with one unit corresponding to the predicted intensity. The first and only initial state of the decoder is the last hidden state of the encoder. The hidden states *h*_*t*_ depicted on Fig. 1 are simplified for visualization but correspond to the hidden states of the forward and backward run. The decoder only performs one interaction since it is not predicting a sequence but rather a scalar value, otherwise in each iteration the context vector and next decoder input would be recalculated using all hidden states from the encoder and the previous output from the decoder. The recurrent layers have the same number of units. The number of units and batch sizes differ in the models generated in this study as they were optimized individually during training (Tables S3, S5, and S10).

### 2.4 Training and testing

The implementation was done in Python (v.3.8.8) with TensorFlow [61] (v. 2.5.0) using the following libraries: Scikit-learn [49] (0.24.1), [62]Pandas [63] (v.1.2.4), Matplotlib [64] (v.3.3.4), Seaborn [65] (v.0.11.1), SciPy [66] (v.1.6.2), and NumPy [67] (v.1.20.1).

Initially, the proof-of-concept models were trained and optimized to determine the model performance with the artificial data as well as its capacity to determine the relevancy of each unique sequence element in the predicted output. For the initial proof-of-concept models, one-hot-encoded inputs were applied. Two formats of sequences were generated with a maximum length of 8 and 40, respectively, and input dimensions of batch size x 8 × 10 and batch size x 40 × 21, respectively. Padding was applied to shorter sequences. An detailed description of data and model performance for proof-of-concept models is found in the supplementary material.

Subsequently, the architecture was used for training and optimizing the models using the full repository dataset after filtering. The inputs for the models were one-hot-encoded peptides sequences with a maximum length of 40 residues, where shorter peptides were padded. Thus, the input matrix has a dimension of batch size x 40 × 21, where the 20 AAs and one padding character are included. The data was split into 5 smaller subsets for K-fold cross-validation, where each subset was used as test dataset once while the remaining dataset was used for training.

To investigate the underlying physicochemical properties that influence the MS1 response for peptides, the attention weights for the 20 AAs were determined. The assigned attention weights for each sequence element were extracted for each intensity prediction. Then, these weights were averaged for each AA, first within the same sequence (in case there are repeated AAs within the peptide sequence) and subsequently across all the sequences. The relevancy of the physicochemical properties was determined by computing the Pearson correlation coefficient (PCC) between the average attention weights of AAs and each of the 566 AAindex1 indices [48], representing a physicochemical property. An AAindex1 index was considered significant if PCC ≤ −0.7 or PCC ≥ 0.7, corresponding to p-values < 1E-3. For the proof-of-concept models, the average attention weights were correlated with the fixed contribution assigned to each element of the corresponding sequence format in a similar manner (see supplementary information).

Once the relevant physicochemical properties were identified, the data was further subset to improve model performance for predicting MS1 intensity. The performance of the final model was compared with the performance obtained with a RF and a RR model, using the same dataset.

Different loss functions were evaluated, however the mean squared error (MSE) [68], [69] was found to work best as the Loss function. The accuracy measurement during model training was done using the mean absolute error (MAE) to observe the distance between real and predicted intensities. Model performance was expressed by the mean absolute percentage error (MAPE) [70], [71] to more clearly depict the unbiased difference between prediction and real values, as the intensity outputs span a large dynamic range. Adam [72] was the optimizer chosen after different optimizers were evaluated, using its default settings which performed better. The models were trained on NVIDIA Quadro T2000 GPU for 5–30 epochs.

Ultimately, the final model performance was benchmarked by comparing with the performance of more classical algorithms; namely a Random Forest (RF) and a Ridge Regression (RR). For RF and RR, the data required additional processing. The input sequence data was converted into tabular data, by generating 840 variables. Each variable corresponds to a combination of the 20 possible AA plus the padding character and the position of the AA in the sequence (from 1 to 40). If an AA is present in a particular position within the sequence, the variable for that particular AA in that position is assigned a value of 1, otherwise is assigned a value of 0.

## 3 Results and discussion

To ensure satisfactory performance of the fundamental architecture, the model was initially developed using artificial datasets with known ground truth (see supplementary material). Overall, the model architecture performed excellently across the four artificial datasets designed, with MAPE < 1% for simple data and/or data with linear correlation for element contribution. For the more complex artificial dataset representing non-linear element contributions and a large dynamic range of values, designed to emulate real data (Proof-of-concept model 4), the MAPE was slightly higher (∼3%) but still displaying highly accurate predictions. In all cases, the model architecture obtained excellent correlation with the ground truth, illustrated by a PCC > 0.98 for predicted and calculated values. Moreover, the attention mechanism successfully identified the elements with the highest contribution to the scalar output (i.e. the value representing the MS1 intensity).

### 3.1 Dataset clean-up and initial model implementation

**Fig. 2:**
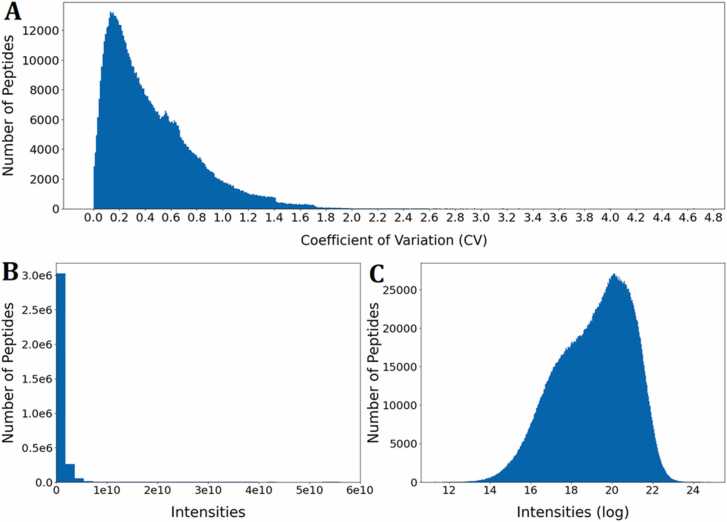
Initial exploration of the cumulated dataset. A. Distribution of coefficient of variation (CV) for peptides with more than one measurement (Bin size ≈ 0.010). B. Distribution for raw MS1 intensities for filtered data (Bin size ≈1.8 ×10^9^. C**.** Distribution of log-transformed MS1 intensity data for filtered data (Bin size ≈ 0.036).

After the model architecture was proven effective using artificial datasets, the model was trained with real data. For this purpose, we extracted the MaxQuant [73] output datafiles from the PROSIT and ProteomicsDB datasets (PRIDE identifiers PXD004732 [46], PXD010595 [34], and PXD021013 [47]), that were produced by Orbitrap analysis of synthetic, equimolar peptide pools. To ensure optimal training of DL models, the noise in the datasets should be reduced. Therefore, we initially inspected the datasets with the aim of investigating variability and data quality. The cumulative database consists of 4016,044 peptide identifications representing 1331,904 unique peptides. As commonly applied in proteomics studies, reverse sequences were eliminated as false positives and potential contaminants removed to improve data reliability. Because the majority of peptides (865,325 or 64.97%) were analyzed and identified in more than one pool, this allowed us to investigate the variability of the MS1 intensity data by computing the coefficient of variation (CV) for the different intensity measurements of the same peptides (Fig. 2A). MS1 intensities show a high variability with CVs exceeding 400% for some peptides. Thus, the CV was used to filter peptides with high variability (CV > 30%) from the initial dataset. In MaxQuant output data, there are additional metrics commonly employed for downstream filtering and processing prior to further analysis. Some metrics relate to quality of identification, such as the posterior error probability (PEP). While PEP is used in the calculation of the peptide/protein score by the MaxQuant built-in search engine Andromeda [74], other factors are also accounted for when calculating this score [75]. The score is directly applied in the filtering during initial MaxQuant analysis through the false discovery rate (FDR), assigned by the user. Consequently, the PEP may also be used as a stand-alone metric to perform further quality-based filtering. As such, we used PEP as a parameter to remove potentially false positive peptides by defining a maximum threshold of 1% (i.e., removing peptides with PEP > 0.01).

The filtered dataset consisted of 320.741 unique peptides for which the MS1 intensity output was log-transformed with the natural logarithm. This reduced the dynamic range of intensity outputs, thereby reducing the impact of high-intensity peaks, and generating a distribution closer to normal (Fig. 2B, C). In addition to noise reduction, the overall effect of filtering was primarily a reduction in size of the dataset without affecting the distribution or dynamic range substantially (Fig. S5).

During initial model training and optimization, two consistent patterns were observed in the obtained attention weights for each of the 20 AAs. While the patterns are quite different, the performance of the different models were comparable, with MAPEs generally in the range from 12% to 17% (see supplementary information). The first pattern frequently highlighted the influence of bulky hydrophobic (i.e., leucine (Leu), isoleucine (Ile), and valine (Val)) and aromatic (i.e., tryptophan (Trp), phenylalanine (Phe), and tyrosine (Tyr)) AAs. In contrast, the second pattern primarily highlighted a high contribution by positively charged AAs (i.e., arginine (Arg) and lysine (Lys), and to a lesser extent histidine (His)). Although these patterns were frequently observed during the training and optimization process, the exact results, namely the attention weights and their distribution, were not consistently reproducible due to the stochastic nature of the algorithm. In other words, the order of the AAs occasionally shuffled, but the overall pattern remained intact. After computing the correlation between the attention weights and the parameters contained in AAindex1, certain physicochemical properties were reproducibly identified for the highly contributing AAs within the two different patterns emerging. To illustrate this, representative models were selected for further analysis.

### 3.2 Representative model 1: Bulky hydrophobic and aromatic amino acids

**Fig. 3:**
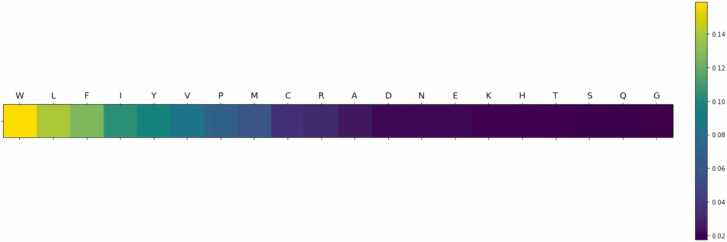
Graphical representation of the attention weights of each AA for representative model 1. The color coding indicates the assigned contribution of each AA to the prediction of the MS1 intensity output from high contribution (yellow) gradually decreasing to low contribution (dark blue).

In the first representative model, the highest attention weights were given mainly to bulky hydrophobic and aromatic AAs (Fig. 3 and Table S6). Trp received the highest attention of all AAs followed by Leu, Phe, and Ile. Tyr received lower attention compared to the other aromatic AAs Furthermore, proline (Pro) and sulphur-containing AAs (i.e., cysteine (Cys) and methionine (Met) also received some attention from the model.

Computing the correlation between AA attention weights and AAindex1, parameters related with hydrophobicity (Tables 1 and S7) were found of significant relevance (p < 5E-4) as indicated by a high PCC and correspondingly low p-values. This indicates a strong correlation between hydrophobicity and the MS1 intensity measurement. That Tyr received the lowest attention of the aromatic AAs can be explained by the hydroxyl group on the aromatic moiety. As hydrophobicity appears to be a key factor, the hydroxyl group increases side chain polarity and thus reduce overall hydrophobicity of the side chain. While Phe is generally considered more hydrophobic than Trp, Trp contains a bulkier side chain and thus overall size/volume, which could indicate bulkiness may be of relevance. But more importantly, Trp is also known to function as a gas-phase charge stabilizer through the indole moiety [76], [77]. This improves stability of the precursor ion, adding to the overall influence on the MS1 response.

**Table 1:** Top 5 relevant physicochemical properties from the AAindex1 identified by correlating the indices with the attention weights of representative model 1.

**Accession number**	**Data description**	**Correlation Score**	**p-value**
MEEJ810102	Retention coefficient in NaH2PO4 (Meek-Rossetti, 1981)	0.94	4.1E-10
MEEJ810101	Retention coefficient in NaClO4 (Meek-Rossetti, 1981)	0.94	8.3E-10
BULH740101	Transfer free energy to surface (Bull-Breese, 1974)	-0.93	1.7E-09
GUOD860101	Retention coefficient at pH 2 (Guo et al., 1986)	0.93	3.0E-09
PARJ860101	HPLC parameter (Parker et al., 1986)	-0.93	4.1E-09

*Retention coefficients and hydrophobicity indices* were often identified as relevant indices. In reverse phase (RP) chromatography with applied solvent gradients going from high towards low polarity, higher peptide retention times are a result of higher peptide hydrophobicity. As acetonitrile, which is commonly used as the organic phase in LC-MS/MS-based proteomics, has a higher vapor pressure than water, it is substantially more volatile. Thus, when peptides with higher retention times (i.e., eluting late) reach the ion source, the solvent is easier to evaporate. Moreover, hydrophobic peptides are generally more inclined to be in the organic phase [78], explaining why *partition coefficient* was another relevant property identified. Furthermore, hydrophobic peptides are usually located towards the surface of the droplets [79], [80], which is also reflected by the identification of different *transfer free energy* properties as relevant. These factors illustrate why more hydrophobic peptides generally have a better ionization efficiency in gradient RP-HPLC. Other studies have found a direct empirical correlation between ionization efficiency and peptide retention times in RP-HPLC, corroborating our findings [78], [80], [81]. While Cys is not considered bulky, the thiol has been alkylated (carbamidomethyl), increasing the size of the side chain substantially. The attention weights were, however, modest, which could be explained by Cys being in the form of carbamidomethylcysteine, increasing the overall polarity of the side chain compared to aliphatic AAs of similar size/volume. The importance of AA size/volume can also explain why alanine (Ala) received substantially lower attention than more bulky hydrophobic AAs (i.e. Val, Leu, and Ile).

Other computational approaches have found results similar to our findings [35], [37], [39], [82], [83]. Jarnuczak et al. (2016) found that in complex mixtures, there is a weak non-linear relationship between ionization efficiency and hydrophobicity, which they argue might be linear in a simpler mixture [84]. The authors also showed that ionization efficiency is hampered at very low and high organic concentration of the mobile phase, as “weak flyers” were observed at both low and high organic concentration of the mobile phase. They state that at very high organic concentrations, there is an increased basicity in acetonitrile within the gas phase, which interferes with the ionization of peptides. Thus, previous studies also indicate that peptide hydrophobicity has an influence on ionization efficiency and thus MS1 response in RP-HPLC-ESI-MS/MS, thereby corroborating our findings.

As the hydrophobicity and retention coefficients were determined to be highly relevant for peptide response, we investigated if this was directly reflected in the filtered dataset. While two indices showed higher correlations with the attention weights from representative model 1 (Table 1), these are retention coefficient in solvents not common employed in ESI-MS. Consequently, we computed the next three indices (BULH740101, GUOD860101, and PARJ860101) for all peptides as both sum and mean and plotted against the peptide MS1 intensity (Fig. S6). No direct correlation was observed and thus, intensity response cannot be predicted based solely on hydrophobicity. While higher responses were observed in certain ranges for the different metrics, these merely represent a higher density of datapoints. Nevertheless, the model identified hydrophobicity as relevant, but the property is not descriptive as a stand-alone variable, and hence the model is finding more complex patterns within the data.

### 3.3 Representative model 2: Positively charged amino acids

**Fig. 4:**
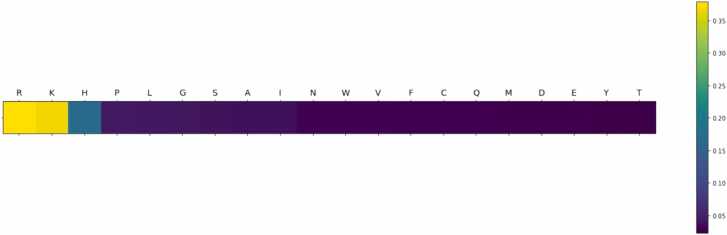
Graphical representation of the attention weights of each AA for representative model 2. The color coding indicates the assigned contribution of each AA to the prediction of the MS1 intensity output from high contribution (yellow) gradually decreasing to low contribution (dark blue).

In the second commonly observed pattern, high relevance of AAs with positively charge side chains (Arg, Lys, and to a lesser extent, His) was observed (Fig. 4 and Table S8). Correlating attention weights with AAindex1, parameters related with peptide charge were, not surprisingly, found to be very important for this model. *Positive charge* and *net charge* had PCCs of 0.93 and 0.74 were found to be statistically significant with p-values of < 2E-09 and < 2E-04, respectively (Table S9). Since the samples were originally analyzed in positive mode ESI-MS using an acidified solvent (0.1% formic acid), that makes *positive charge* a very intuitive property. The parameter precisely points to those AAs that most likely will be positively charged due to side chain protonation at acidic pH. Thus, the presence of Arg, Lys, and His in a peptide most likely will increase the probability of getting a positively charged ion during ionization. Other studies have also found this particular property of high relevance [39], [84], [85].

To investigate if the relevance of positive charge was directly reflected in the filtered dataset, the number of positively charged AAs (Arg, Lys, and His), net charge at pH 7, and net charge at pH 3 (reflecting the acidic environment used during positive mode ESI-MS) was determined for individual peptides and plotted against MS1 intensity (Fig. S7A-D). Moreover, these charge-related metrics were also determined in a length-normalized version (charge/length) to investigate the interplay between the two physicochemical properties (Fig. S7E-F)). As found for hydrophobicity descriptors in relation to representative model 1, there was no direct correlation between charge and MS1 intensity, also indicating a more complex interplay between different variables, which the model is able to identify. We also investigated different combinations of hydrophobicity indices and charge (i.e., ratios and products), but also here found these metrics insufficient to describe MS1 intensity (data not shown).

#### 3.3.1 Sub-distributions and search parameter-based data subsetting

The distribution of the log transformed MS1 intensities in the filtered dataset (Fig. 2C) was to no extent normally distributed and appeared to contain more than one distribution. To investigate this, the dataset was subset according to variable parameters in the MaxQuant metadata related to specified enzymatic digestion and search parameters. When grouping the data based on the specified enzyme and the enzyme mode (i.e., specific, semi-specific, or unspecific *in silico* digestion) [34], [46], [47], the presence of sub-distributions was evident.

**Fig. 5:**
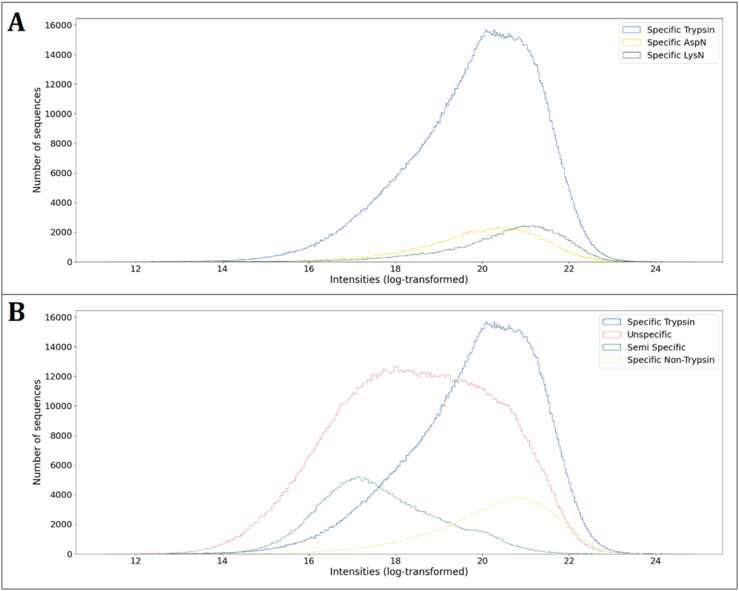
Histograms of log-transformed peptide MS1 intensity outputs by “Enzyme” and “Enzyme Mode”. A. Histogram of peptides quantified using “specific digestion”. B. Histogram with peptides grouped by MaxQuant “enzyme mode” and distinguishing between tryptic or non-tryptic peptides using “specific digestion”.

Peptides searched with a specific enzyme digestion (Trypsin, LysN, and AspN) displayed a higher median value of intensity than peptides searched with unspecific or semi-specific digestion (Fig. 5A, B and Table 2). Trypsin generates peptides with a C-terminus constituted by Arg or Lys, LysN produces peptides with a N-terminal Lys, while AspN releases peptides with an N-terminal aspartic acid (Asp). While all these specific terminal AAs have charged side chains, Arg and Lys are positively charged while Asp is negatively charged. Distribution of log transformed MS1 intensities seem to suggest that charged AAs, especially when located at the peptide termini, may have a direct effect on the intensity output in MS1. However, Asp was not identified as high relevance (Fig. 4). Asp will not be charged under acidic pH used in positive mode ESI-MS, and therefore constitute an unchanged, polar residue. As such, the higher median intensity of this subset may reflect a potential proximity effect of carboxylic acid moiety and the N-terminal charged amine but may also represent that the peptide composition in the subset is indigenously more suitable for MS detection and hence provides higher MS1 response.

**Table 2:** Median values of log-transformed intensities and mean length of peptides (whole dataset) grouped by “Enzyme Mode” and “Enzyme” specified in MaxQuant metadata.

**Enzyme Mode/Enzyme**	**Median of Log-transformed Intensities**	**Mean Length**
Specific/Trypsin	20.03	13.25
Specific/AspN	20.13	14.79
Specific/LysN	20.76	14.87
Unspecific	18.05	11.10
Semi specific	17.05	19.00

It is important to highlight that peptides searched with semi-specific setting may have shown lower intensity values (Fig. 5B) for two reasons. Firstly, the pools used for these analyses contained longer peptide (average > 25 AAs [47]) than in the other datasets, which can generate a reduction of the intensity measurement due to a bias against longer peptides in the orbitrap mass analyzer [84], [86], [87], [88]. Secondly, the search mode acilitated identification of full-length synthetic peptides as well as truncations obtained as incomplete synthesis products [47]. As the pool equimolarity correspond to the full-length peptide, the abundance of truncated forms is expected in substantially lower, thus reducing the intensity values of the detected truncated sequences (Fig. S8C, D). This directly compromises the equimolar prerequisite for the sequence-centric analysis performed in this study and ultimately introduce bias and reduced reliability of the dataset. This becomes particularly evident through the mean length of the identified peptides using semi-specific searches (Table 2), as this (19 AAs) is substantially lower than the reported average length for the peptide pools (> 25 AAs). For unspecific *in silico* digestion (Fig. 5B), there does not clearly seem to be higher response for peptides with a C-terminal Arg or Lys, although tryptic peptides identified in unspecific searches do represent the high responders, too (Fig. S9A). The apparent bimodal distribution indicates that additional properties account for the segregation of this subset in to (at least) two additional subsets. Interestingly, there seems to be a more consistent increase in MS1 intensity for peptides containing Arg or Lys (anywhere in the sequence) in comparison to those that do not (Fig. S9B). This observation further substantiates the importance of positively charged AAs for an increased ionization efficiency and thus MS1 response (thereby corroborating the findings from representative model 2), while length itself does not seem to correlate directly with MS1 response in general terms (Fig. S8A, C). While length does seem to influence response to some degree, this may simply be related to the fact that these lengths are in general overrepresented in the dataset (Fig. S8B, D).

### 3.4 The influence of structural properties on the peptide level

In addition to hydrophobicity and charge, a number of physicochemical properties were identified as relevant in the two representative models, which relate to protein and peptide structural properties (Tables S7 and S9). These parameters were found with a high PCC and p-values considerably lower than 0.05. Although many of these properties are also related to e.g., hydrophobicity, they also contain information on structural aspects, as these are often related. For instance, one of the properties showing high correlation with attention weights is the *Atom-based hydrophobic moment.* This parameter quantifies the strength of the periodicity in the polar or hydrophobic nature of the constituent amino acids of a sequence, which is related to the stability and type of structure as well as its functions [89]. Other properties such as *Entropy of formation, solvation free energy,* and *Weights from the IFH scale* were also found relevant. These properties are related to the thermodynamics of protein and peptide conformation and stability [90], [91], [92]. *Energy transfer from out to in (95%buried)* and *Buriability “provides a quantitative measure of the driving force for the burial of a residue”*, thereby describing polarity-driven, tertiary conformational properties [93]. While the *Isoelectric point* is a parameter related to charge, it also describes electrostatic interactions between AA side chains, which affect protein and peptide structure [94], [95].

There were, however, also important properties identified that more directly relate to structural aspects of peptides and proteins. For instance, the *Helix termination parameter at position j-2,j-1,j* refers to the formation probability of secondary structures, here specifically α-helices, in peptides [95]. Peptides and proteins can form secondary and tertiary structures not only in solution, but also in the gas phase [96], [97], [98]. Studies have shown that peptides with stable α-helical and β-sheet structures in solution have lower intensity response than corresponding structurally disturbed analog peptides (L- to D-AA substitution) in MALDI-MS [99]. This indicates that peptide solution-phase structure has a significant influence on the MS1 response. Moreover, it has been observed that the fragmentation of protonated peptides is influenced by the peptide’s gas-phase secondary structure and in particular acid-base interactions and charge solvation in the gas phase [100]. This substantiates that proximity-based intramolecular interactions are indeed of importance for precursor stability during MS analysis, why peptides with N-terminal Asp (AspN) were generally found to show high median intensities (Table 2). Consequently, the identification of a peptide is influenced by both the peptide primary structure and the consequential secondary structure in the gas phase. In-source fragmentation would lead not only to a lower MS1 response, but also a reduced proportion of the precursor peptide available for MS/MS identification. Furthermore, studies using MALDI-MS have shown that the conformation of peptides in the gas-phase is not necessarily the same than in solution-phase [101]. While ionization method in these studies differs from ESI considered here, the phase transition is still highly relevant and considered of importance in relation to ionization efficiency and thus peptide MS1 response in ESI-MS/MS. Moreover, other studies with computational approaches have similarly found structural properties of significant relevance for MS analysis [35], [37], [39], [83], [84]. Based on these findings, peptide structure appears a key factor affecting the MS1 response and an important source of variability in intensity measurements.

### 3.5 Model performance optimization and sequence-based intensity prediction

The presented models were evaluated with the test datasets and their performances were expressed through MAPE, showing the percentual distance between the real and predicted MS output intensities. The proof-of-concept models displayed a MAPE between 0.56% and 3.2% (Tables S3 and S5) with an almost perfect correlation between expected and predicted values (Figs. S2 and S4) with p-values < 1E-5 and as low as 3E-23 for proof-of-concept model 3 (Tables S3 and S5). This shows that the models have an exceptional performance with the artificial data, not only identifying the average contribution of each unique elements of the sequence but also predicting the expected output. Using the repository MS data, initially all the filtered data was used to train and test the two representative models, obtaining an average MAPE of 14.8% for log-transformed intensities (Table S10). The low standard deviations (< 0.5%) from the 5-fold cross validation show that the model architecture is capable of reproducibly finding descriptive patterns in the data. Nevertheless, there are substantial differences in intensity distributions based on the applied enzyme and enzyme mode setting used during the data search (Fig. 5B). Therefore, to improve the model performance, the model was trained and tested only using a specific subset of the data, namely the Specific/Tryptic peptides, as the remaining subsets had substantial uncertainties, as previously discussed.

**Fig. 6:**
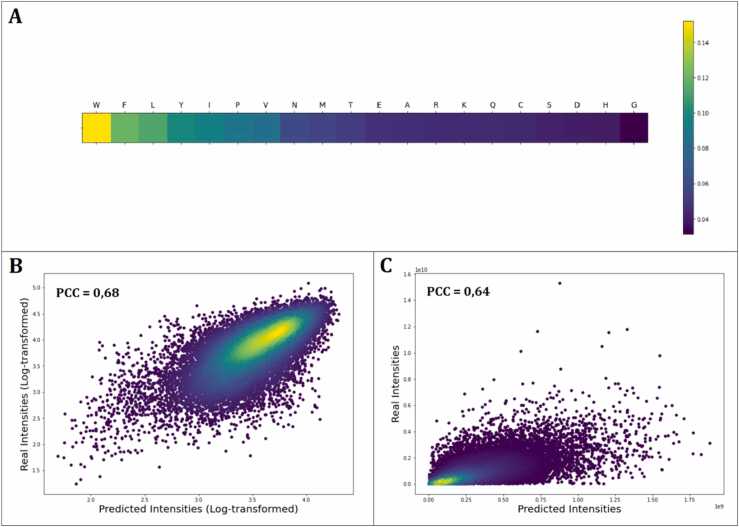
Attention weights and model performance results for the final mode (Specific/Tryptic peptides only). A. Graphical representation of the attention weights of individual AAs. The color coding indicates the assigned contribution of each AA to the MS1 intensity output given by the model. **B.** Scatter and density plot of the measured vs the predicted intensities (log-transformed). **C.** Scatter and density plot of the measured vs the predicted intensities (real scale).

When doing this, the MAPE was reduced to 9.7% (Table S10), resulting in a relative reduction in the error of 35% for log-transformed intensities but also an impressive 56% for raw intensities (MAPE=98%) compared to the average MAPEs for the two representative models (average MAPE=219%). Moreover, when comparing the performance of the final model against random forest and ridge regression models, we observe similar MAPEs for the log-transformed intensities, but our model has higher significantly PCC for the log-transformed (PCC = 0.68) and real scale (PCC = 0.64) predictions as well as a much lower MAPE for real scale predictions (Table 3 and Fig. 6B, C). Thus, this indicates that our model has a better performance than the more classical algorithms used for benchmarking in this study, as the predictions made by our model seem to be more adjusted to real values (higher PCC), and thus more translatable to real scale values.

**Table 3:** Performance metrics (expressed as MAPE (%) and PCC on real and log-transformed scale) for the final model on the specific/tryptic data subset. For benchmarking of the model performance, random forest and ridge regression models were included for comparison. All metrics represent average ± standard deviation for 5-fold cross validation.

	Log-Transformed Data		Real Scale Data	
Model	MAPE^a^ (%)	PCC^b^	MAPE^a^ (%)	PCC^b^
Encoder-decoder with attention mechanism (Final model)	9.67 ± 0.53	0.68 ± 0.01	97.5 ± 6.2	0.64 ± 0.01
Random Forest	9.09 ± 0.07	0.57 ± 0.01	251,4 ± 16.4	0.56 ± 0.01
Ridge Regression	9.19 ± 0.08	0.54 ± 0.01	269.3 ± 18.6	0.55 ± 0.01

a Mean absolute percentage error.

b Pearson correlation coefficient.

The attention weights of the final model consistently focused on the bulkier hydrophobic and aromatic AAs (Fig. 6A and Table S11), thereby showing similar attention patterns as representative model 1 did for the whole filtered dataset, and thus giving higher relevance to the hydrophobicity-related properties from AAindex1 (Table S12). This shows that the transition of peptides from liquid to gas phase and charge stabilization are key factors in sequence-based variability for MS1 intensity measurements. Moreover, this observation is likely a result of further dataset segmentation meaning that the model is now focusing on these properties as all the peptides in the particular data subset are tryptic. As all peptides feature a C-terminal Arg/Lys, charge may not be of descriptive relevance. In turn, this indicates that representative model 2 in fact focus more on identification of the specific/tryptic subset, as these peptides overall show a higher MS1 intensity compared to the semi-specific and unspecific subsets (Table 2). To investigate this further, we determined charge-related metrics for these subset peptides and investigated the correlation with the intensity outputs (Fig. S10). Here we found that neither charge nor number of positively charged AAs seem to in any way be descriptive of MS1 intensity variation between peptides, as observed in the previous models. Moreover, the relationship between MAPE (%) of each prediction and peptide length was investigated showing no correlation, indicating that the model had no bias regarding peptide length (Fig. S11).

When evaluating model performance, it is important to take into consideration that the models were trained only providing the sequence information and the corresponding MS1 intensity output without explicitly defining any physicochemical properties to be important. Nevertheless, the models identified certain underlying properties by themselves, which align with previous empirical studies. Furthermore, as there was a clear correlation between predicted and real MS1 intensity outputs (both raw and log-transformed), this shows that the models are effectively extracting meaningful information from the peptide sequences to predict intensity. Nonetheless, there is a limit to how much the information from the sequences can explain the MS1 intensity output, since there are other sources of variability. Such limitation arise from sources such reproducibility in sampling and sample preparation [102], [103], [104], the type of MS technology employed [1], [84], [104], [105], [106], [107], as well as the pipeline used for raw data processing [108], [109]. Moreover, it is essential to consider that there is high variability in the MS1 intensity output for the same peptides across different pools within this particular dataset. Such variability is highly affected by the competition for ionization between co-eluting peptides [110], [111]. While co-elution is generally considered a major concern in MS2 and thus for peptide identification, particularly in data-dependent acquisition, it may still be a potential source of variability in MS1. Peptide identification rates may be further improved by e.g. expanding the model to independent acquisition strategies such as DIA [112], SWATH [113], [114] or BoxCar [115]. A potential way for alleviating the problem while directly reducing co-elution, thereby improving quality of MS1 response data, could be to look towards longer gradients and particularly pre-MS1 separation by ion mobility [116], [117]. The presented model architecture in this work does not explicitly account for co-elution and the effect on MS1 response, however, by using median MS1 intensities across multiple pools, input data reflects a more “average state” for each peptide. In future development of peptide-level quantitative models, this could be investigated and potentially dealt with by implementation of modules that can predict peptide co-elution through e.g. retention time prediction [34], [118]. Consequently, building more robust datasets and designing standardized experimental protocols that allows to generate more consistent measurements are key factors to building models that can accurately predict peptide MS1 intensities and account for intrinsic variability. Such models can ultimately be applied to estimate absolute peptide quantification without the need of isotopically labeled surrogate peptides and illustrates potential for developing fundamentally new approaches within the field of label-free BUP.

## 4 Conclusions

In this study, a deep learning neural network with attention mechanism was used to determine the relevance of each of the 20 natural amino acids on the MS1 signal response from peptides in HPLC-ESI-MS/MS analysis of equimolar peptide pools. The initial models were capable of predicting log-transformed peptide intensity with an average MAPE of 14.8%. The attention weights from the models were correlated with the physicochemical property indices contained in AAindex1 to identify which physicochemical properties play an important role in the behavior of peptides in MS, as well as their impact on MS1 intensity measurements. Hydrophobicity, charge, and peptide gas-phase structure were identified as important relevant properties governing the peptide MS1 responses. These parameters were not directly reflected in the data, but extractable using the presented model architecture through the inclusion of an attention mechanism. Following further segmentation of the dataset, the model was trained on only specific/tryptic peptides, thereby improving the model performance, and reducing MAPE for log intensity prediction to 9.7%. The model showed high reproducibility through K-fold cross-validation and overall outperformed classical random forest and ridge regression models. The model performance is likely to be improved by generating more accurate and robust datasets as well as experimental protocols to normalize between individual MS runs. Overall, the information generated in this study is of great relevance to understand the key factors influencing the results obtained in HPLC-ESI-MS/MS peptide analysis. This understanding can also be used to build more advanced models for peptide detectability and peptide quantification and may ultimately find use for development of new protein-level quantification strategies in label-free proteomics.

## CRediT authorship contribution statement

**Naim Abdul-Khalek:** Conceptualization, Methodology, Software, Validation, Formal analysis, Investigation, Data curation, Writing − original draft, Writing − review & editing, Visualization. **Reinhard Wimmer:** Conceptualization, Resources, Writing − review & editing, Supervision. **Michael Toft Overgaard:** Conceptualization, Resources, Writing − review & editing, Supervision, Funding acquisition. **Simon Gregersen Echers:** Conceptualization, Methodology, Resources, Validation, Investigation, Writing − original draft, Writing − review & editing, Supervision, Project administration, Funding acquisition.

## Declaration of Competing Interest

The authors declare no conflict of interest.

## Data Availability

The data used in this project can be found in the PRIDE repository with the identifiers PXD004732, PXD010595, and PXD021013 including the mass spectrometric raw data and the search data.

## References

[1] Awad H., Khamis M.M., El-Aneed A. Mass Spectrometry, Review of the Basics: Ionization. 2014;50:158–75. 10.1080/05704928.2014.954046.

[2] Herrero M., Simõ C., García-Cañas V., Ibáñez E., Cifuentes A. (2012). Foodomics: MS-based strategies in modern food science and nutrition. Mass Spectrom Rev.

[3] Davison J., O’Gorman A., Brennan L., Cotter D.R. (2018). A systematic review of metabolite biomarkers of schizophrenia. Schizophr Res.

[4] Hofstadler S.A., Sannes-Lowery K.A. (2006). Applications of ESI-MS in drug discovery: interrogation of noncovalent complexes. Nat Rev Drug Discov.

[5] García-Moreno P.J., Gregersen S., Nedamani E.R., Olsen T.H., Marcatili P., Overgaard M.T. (2020). Identification of emulsifier potato peptides by bioinformatics: application to omega-3 delivery emulsions and release from potato industry side streams. Sci Rep.

[6] Gregersen S., Kongsted A.S.H., Nielsen R.B., Hansen S.S., Lau F.A., Rasmussen J.B. (2021). Enzymatic extraction improves intracellular protein recovery from the industrial carrageenan seaweed Eucheuma denticulatum revealed by quantitative, subcellular protein profiling: A high potential source of functional food ingredients. Food Chem X.

[7] El-Aneed A., Cohen A., Banoub J. (2009). Mass spectrometry, review of the basics: electrospray, MALDI, and commonly used mass analyzers. Appl Spectrosc Rev.

[8] Wilm M. (2011). Principles of Electrospray Ionization. Mol Cell Proteom.

[9] Liuni P., Wilson D.J. Understanding and optimizing electrospray ionization techniques for proteomic analysis. 2014;8:197–209. 10.1586/EPR.10.111. 21501013

[10] Cañas Montalvo B., López-Ferrer D., Ramos-Fernández A., Camafeita E., Calvo E. (2006). Mass spectrometry technologies for proteomics. Brief Funct Genom.

[11] Schwanhüusser B., Busse D., Li N., Dittmar G., Schuchhardt J., Wolf J. (2011). Global quantification of mammalian gene expression control. Nature.

[12] Cox J., Hein M.Y., Luber C.A., Paron I., Nagaraj N., Mann M. (2014). Accurate proteome-wide label-free quantification by delayed normalization and maximal peptide ratio extraction, termed MaxLFQ. Mol Cell Proteom.

[13] Nikolov M., Schmidt C., Urlaub H. (2012). Quantitative mass spectrometry-based proteomics: An overview. Methods Mol Biol.

[14] Xie F., Liu T., Qian W.J., Petyuk V.A., Smith R.D. (2011). Liquid Chromatography-Mass Spectrometry-based Quantitative Proteomics *. J Biol Chem.

[15] Vidova V., Spacil Z. (2017). A review on mass spectrometry-based quantitative proteomics: Targeted and data independent acquisition. Anal Chim Acta.

[16] Nahnsen S., Bielow C., Reinert K., Kohlbacher O. Tools for Label-free Peptide Quantification* □ S, 2012. 10.1074/mcp.R112.025163. PMC3591650 23250051

[17] He B., Shi J., Wang X., Jiang H., Zhu H.J. (2019). Label-free absolute protein quantification with data-independent acquisition. J Proteom.

[18] Wiśniewski J.R., Hein M.Y., Cox J., Mann M.A. (2014). “proteomic ruler” for protein copy number and concentration estimation without spike-in standards. Mol Cell Proteom.

[19] Jafarpour A., Gregersen S., Gomes R.M., Marcatili P., Olsen T.H., Jacobsen C. (2020). Biofunctionality of Enzymatically Derived Peptides from Codfish (Gadus morhua) Frame: Bulk In Vitro Properties, Quantitative Proteomics, and Bioinformatic Prediction. Mar Drugs.

[20] Gregersen Echers S., Jafarpour A., Yesiltas B., García-Moreno P.J., Greve-Poulsen M., Hansen D.K. (2023). Targeted hydrolysis of native potato protein: A novel workflow for obtaining hydrolysates with improved interfacial properties. Food Hydrocoll.

[21] Millikin R.J., Solntsev S.K., Shortreed M.R., Smith L.M. (2018). Ultrafast peptide label-free quantification with FlashLFQ. J Proteome Res.

[22] Blein-Nicolas M., Zivy M. (2016). Thousand and one ways to quantify and compare protein abundances in label-free bottom-up proteomics. Biochim Et Biophys Acta (BBA) - Proteins Proteom.

[23] Daly D.S., Anderson K.K., Panisko E.A., Purvine S.O., Fang R., Monroe M.E. (2008). Mixed-effects statistical model for comparative LC-MS proteomics studies. J Proteome Res.

[24] Wen B., Zeng W.-F., Liao Y., Shi Z., Savage S.R., Jiang W. (2020). Deep Learning in Proteomics. Proteomics.

[25] Chollet F. (2017). Deep Learning with Python.

[26] Alquraishi M. (2019). AlphaFold at CASP13. Bioinformatics.

[27] Sun T., Zhou B., Lai L., Pei J. (2017). Sequence-based prediction of protein protein interaction using a deep-learning algorithm. BMC Bioinforma.

[28] Kulmanov M., Hoehndorf R. (2020). DeepGOPlus: improved protein function prediction from sequence. Bioinformatics.

[29] Meyer J.G. (2021). Deep learning neural network tools for proteomics. Cell Rep Methods.

[30] Sonsare P.M., Gunavathi C. (2019). Investigation of machine learning techniques on proteomics: A comprehensive survey. Prog Biophys Mol Biol.

[31] Xu C.M., Zhang J.Y., Liu H., Sun H.C., Zhu Y.P., Xie H.W. (2010). Advance of peptide detectability prediction on mass spectrometry platform in proteomics. Chin J Anal Chem.

[32] Sutskever I., Vinyals O., Le Q.V. (2014). https://arxiv.org/abs/1409.3215v3.

[33] Sehovac L., Grolinger K. (2020). Deep Learning for Load Forecasting: Sequence to Sequence Recurrent Neural Networks with Attention. IEEE Access.

[34] Gessulat S., Schmidt T., Zolg D.P., Samaras P., Schnatbaum K., Zerweck J. (2019). Prosit: proteome-wide prediction of peptide tandem mass spectra by deep learning. Nat Methods.

[35] Gao Z., Chang C., Yang J., Zhu Y., Fu Y. (2019). AP3: an advanced proteotypic peptide predictor for targeted proteomics by incorporating peptide digestibility. Anal Chem.

[36] Riley R.M., Miko S.E.S., Morin R.D., Morin G.B., Negri G.L. (2023). PeptideRanger: An R Package to Optimize Synthetic Peptide Selection for Mass Spectrometry Applications. J Proteome Res.

[37] Eyers C.E., Lawless C., Wedge D.C., Lau K.W., Gaskell S.J., Hubbard S.J. (2011). CONSeQuence: prediction of reference peptides for absolute quantitative proteomics using consensus machine learning approaches. Mol Cell Proteom.

[38] Pauletti B.A., Granato D.C., M. Carnielli C., Câmara G.A., Normando A.G.C., Telles G.P. (2023). Typic: A Practical and Robust Tool to Rank Proteotypic Peptides for Targeted Proteomics. J Proteome Res.

[39] Zimmer D., Schneider K., Sommer F., Schroda M., Mühlhaus T. (2018). Artificial intelligence understands peptide observability and assists with absolute protein quantification. Front Plant Sci.

[40] Rusilowicz M., Newman D.W., Creamer D.R., Johnson J., Adair K., Harman V.M. (2023). AlacatDesigner─computational design of peptide concatamers for protein quantitation. J Proteome Res.

[41] Mallick P., Schirle M., Chen S.S., Flory M.R., Lee H., Martin D. (2006). Computational prediction of proteotypic peptides for quantitative proteomics. Nat Biotechnol.

[42] Demeure K., Duriez E., Domon B., Niclou S.P. (2014). Peptide manager: A peptide selection tool for targeted proteomic studies involving mixed samples from different species. Front Genet.

[43] Chen Q., Jiang Y., Ren Y., Ying M., Lu B. (2020). Peptide Selection for Accurate Targeted Protein Quantification via a Dimethylation High-Resolution Mass Spectrum Strategy with a Peptide Release Kinetic Model. ACS Omega.

[44] Vaudel M., Burkhart J.M., Zahedi R.P., Oveland E., Berven F.S., Sickmann A. (2015). PeptideShaker enables reanalysis of MS-derived proteomics data sets. Nat Biotechnol.

[45] Rehfeldt T.G., Krawczyk K., Bøgebjerg M., Schwammle V., Rottger R. (2022). MS2AI: automated repurposing of public peptide LC-MS data for machine learning applications. Bioinformatics.

[46] Zolg D.P., Wilhelm M., Schnatbaum K., Zerweck J., Knaute T., Delanghe B. (2017). Building ProteomeTools based on a complete synthetic human proteome. Nat Methods.

[47] Wilhelm M., Zolg D.P., Graber M., Gessulat S., Schmidt T., Schnatbaum K. (2021). Deep learning boosts sensitivity of mass spectrometry-based immunopeptidomics. Nat Commun.

[48] Kawashima S., Ogata H., Kanehisa M. (1999). AAindex: amino acid index database. Nucleic Acids Res.

[49] Pedregosa F., Varoquaux G., Gramfort A., Michel V., Thirion B., Grisel O. (2012). Scikit-learn: Machine Learning in Python. J Mach Learn Res.

[50] Liu K., Li S., Wang L., Ye Y., Tang H. (2020). Full-spectrum prediction of peptides tandem mass spectra using deep neural network. Anal Chem.

[51] Silva A.S.C., Bouwmeester R., Martens L., Degroeve S. (2019). Accurate peptide fragmentation predictions allow data driven approaches to replace and improve upon proteomics search engine scoring functions. Bioinformatics.

[52] Zhou C., Bowler L.D., Feng J. (2008). A machine learning approach to explore the spectra intensity pattern of peptides using tandem mass spectrometry data. BMC Bioinforma.

[53] Bowden P., Thavarajah T., Zhu P., McDonell M., Thiele H., Marshall J.G. (2012). Quantitative statistical analysis of standard and human blood proteins from liquid chromatography, electrospray ionization, and tandem mass spectrometry. J Proteome Res.

[54] Ryu S., Goodlett D.R., Noble W.S., Minin V.N. A statistical approach to peptide identification from clustered tandem mass spectrometry data. 2012 IEEE International Conference on Bioinformatics and Biomedicine Workshops, 2012:648–653. 10.1109/BIBMW.2012.6470214. PMC3698614 23828149

[55] Chung J., Gulcehre C., Cho K., Bengio Y. Empirical Evaluation of Gated Recurrent Neural Networks on Sequence Modeling, 2014. 10.48550/arxiv.1412.3555.

[56] Bahdanau D., Cho K.H., Bengio Y. Neural Machine Translation by Jointly Learning to Align and Translate. 3rd International Conference on Learning Representations, ICLR 2015 - Conference Track Proceedings, 2014. 10.48550/arxiv.1409.0473.

[57] Pascanu R., Mikolov T., Bengio Y. On the difficulty of training recurrent neural networks. Proceedings of the 30th International Conference on Machine Learning, vol. 28, PMLR; 2013, p. 1310–1318. 10.48550/arXiv.1211.5063.

[58] Gu J., Lu Z., Li H., Li V.O.K. (2016).

[59] Ayoub S., Gulzar Y., Reegu F.A., Turaev S. (2022). Generating Image Captions Using Bahdanau Attention Mechanism and Transfer Learning. Symmetry (Basel).

[60] Bahdanau D., Cho K.H., Bengio Y. (2015). Neural Machine Translation by Jointly Learning to Align and Translate. International Conference on Learning Representations, International Conference on Learning Representations, ICLR.

[61] Abadi M., Barham P., Chen J., Chen Z., Davis A., Dean J., et al. TensorFlow: A system for large-scale machine learning. Proceedings of the 12th USENIX Symposium on Operating Systems Design and Implementation, OSDI 2016, 2016:265–83. 10.48550/arxiv.1605.08695.

[62] Seabold S., Perktold J. Statsmodels: Econometric and Statistical Modeling with Python. Proceedings of the 9th Python in Science Conference, 2010:92–6. 10.25080/MAJORA-92BF1922-011.

[63] McKinney W. Data Structures for Statistical Computing in Python. Proceedings of the 9th Python in Science Conference, 2010:56–61. 10.25080/MAJORA-92BF1922-00A.

[64] Hunter J.D. (2007). Matplotlib: A 2D graphics environment. Comput Sci Eng.

[65] Waskom M.L. (2021). seaborn: statistical data visualization. J Open Source Softw.

[66] Virtanen P., Gommers R., Oliphant T.E., Haberland M., Reddy T., Cournapeau D. (2020). SciPy 1.0: Fundamental algorithms for scientific computing in Python. Nat Methods.

[67] Harris C.R., Millman K.J., van der Walt S.J., Gommers R., Virtanen P., Cournapeau D. (2020). Array Programming with NumPy. Nature.

[68] Shimobaba T., Kakue T., Ito T. Convolutional Neural Network-Based Regression for Depth Prediction in Digital Holography. 2018 IEEE 27th International Symposium on Industrial Electronics, 2018:1323–6. 10.1109/ISIE.2018.8433651.

[69] Park A., Joo M., Kim K., Son W.J., Lim G.T., Lee J. (2022). A comprehensive evaluation of regression-based drug responsiveness prediction models, using cell viability inhibitory concentrations (IC50 values). Bioinformatics.

[70] Nguyen M., Jankovic I., Kalesinskas L., Baiocchi M., Chen J.H. (2021). Machine learning for initial insulin estimation in hospitalized patients. J Am Med Inform Assoc.

[71] Ren Y., Li X., Xu H. (2022). A deep learning model to extract ship size from Sentinel-1 SAR images. IEEE Trans Geosci Remote Sens.

[72] Kingma D.P., Ba J.L. Adam: A Method for Stochastic Optimization. 3rd International Conference on Learning Representations, 2014. 10.48550/arxiv.1412.6980.

[73] Cox J., Mann M. (2008). MaxQuant enables high peptide identification rates, individualized p.p.b.-range mass accuracies and proteome-wide protein quantification. Nat Biotechnol.

[74] Cox J., Neuhauser N., Michalski A., Scheltema R.A., Olsen J.V., Mann M. (2011). Andromeda: A peptide search engine integrated into the MaxQuant environment. J Proteome Res.

[75] Gregersen S., Pertseva M., Marcatili P., Holdt S.L., Jacobsen C., García-Moreno P.J. (2022). Proteomic characterization of pilot scale hot-water extracts from the industrial carrageenan red seaweed Eucheuma denticulatum. Algal Res.

[76] Weinkauf R., Schanen P., Yang D., Soukara S., Schlag E.W. (1995). Elementary Processes in Peptides: Electron Mobility and Dissociation in Peptide Cations in the Gas Phase. J Phys Chem.

[77] Marchese R., Grandori R., Carloni P., Raugei S. (2010). On the Zwitterionic Nature of Gas-Phase Peptides and Protein Ions. PLoS Comput Biol.

[78] Cech N.B., Krone J.R., Enke C.G. (2001). Predicting electrospray response from chromatographic retention time. Anal Chem.

[79] Cech N.B., Enke C.G. (2000). Relating electrospray ionization response to nonpolar character of small peptides. Anal Chem.

[80] Osaka I., Takayama M. (2014). Influence of hydrophobicity on positive- and negative-ion yields of peptides in electrospray ionization mass spectrometry. Rapid Commun Mass Spectrom.

[81] Vreeke G.J.C., Lubbers W., Vincken J.P., Wierenga P.A. (2022). A method to identify and quantify the complete peptide composition in protein hydrolysates. Anal Chim Acta.

[82] Muntel J., Boswell S.A., Tang S., Ahmed S., Wapinski I., Foley G. (2015). Abundance-based classifier for the prediction of mass spectrometric peptide detectability upon enrichment (PPA). Mol Cell Proteom.

[83] Qeli E., Omasits U., Goetze S., Stekhoven D.J., Frey J.E., Basler K. (2014). Improved prediction of peptide detectability for targeted proteomics using a rank-based algorithm and organism-specific data. J Proteom.

[84] Jarnuczak A.F., Lee D.C.H., Lawless C., Holman S.W., Eyers C.E., Hubbard S.J. (2016). Analysis of intrinsic peptide detectability via integrated label-free and SRM-based absolute quantitative proteomics. J Proteome Res.

[85] Abaye D.A., Pullen F.S., Nielsen B. v (2011). Peptide polarity and the position of arginine as sources of selectivity during positive electrospray ionisation mass spectrometry. Rapid Commun Mass Spectrom.

[86] Gautier V., Boumeester A.J., Lössl P., Heck A.J.R. (2015). Lysine conjugation properties in human IgGs studied by integrating high-resolution native mass spectrometry and bottom-up proteomics. Proteomics.

[87] Searle B.C., Egertson J.D., Bollinger J.G., Stergachis A.B., MacCoss M.J. (2015). Using Data Independent Acquisition (DIA) to Model High-responding Peptides for Targeted Proteomics Experiments. Mol Cell Proteom.

[88] Mallick P., Schirle M., Chen S.S., Flory M.R., Lee H., Martin D. (2006). Computational prediction of proteotypic peptides for quantitative proteomics. Nat Biotechnol.

[89] Eisenberg D., Weiss R.M., Terwilliger T.C. (1984). The hydrophobic moment detects periodicity in protein hydrophobicity. Proc Natl Acad Sci USA.

[90] Doig A.J., Sternberg M.J.E. (1995). Side-chain conformational entropy in protein folding. Protein Sci.

[91] Eisenberg D., Mclachlan A.D. (1986). Solvation energy in protein folding and binding. Nature.

[92] Jacobs R.E., White S.H. (1989). The nature of the hydrophobic binding of small peptides at the bilayer interface: Implications for the insertion of transbilayer helices. Biochemistry.

[93] Zhou H., Zhou Y. (2004). Quantifying the effect of burial of amino acid residues on protein stability. Protein: Struct, Funct, Bioinforma.

[94] Novák P., Havlíček V. Protein Extraction and Precipitation. Proteomic Profiling and Analytical Chemistry: The Crossroads: Second Edition, 2016:51–62. 10.1016/B978-0-444-63688-1.00004-5.

[95] Finkelstein A.V., Badretdinov A.Y., Ptitsyn O.B. (1991). Physical reasons for secondary structure stability: alpha-helices in short peptides. Proteins.

[96] Marcoux J., Robinson C.V. (2013). Twenty years of gas phase structural biology. Structure.

[97] Loo J.A. (1998). Studying noncovalent protein complexes by electrospray ionization mass spectrometry. Mass Spectrom Rev.

[98] Chin W., Compagnon I., Dognon J.P., Canuel C., Piuzzi F., Dimicoli I. (2005). Spectroscopic evidence for gas-phase formation of successive β-turns in a three-residue peptide chain. J Am Chem Soc.

[99] Wenschuh H., Halada P., Lamer S., Jungblut P., Krause E. (1998). The Ease of Peptide Detection by Matrix-assisted Laser Desorption/Ionization Mass Spectrometry: the Effect of Secondary Structure on Signal Intensity. Rapid Commun Mass Spectrom.

[100] Tsaprailis G., Nair H., Somogyi Á., Wysocki V.H., Zhong W., Futrell J.H. (1999). Influence of secondary structure on the fragmentation of protonated peptides. J Am Chem Soc.

[101] Ruotolo B.T., Verbeck G.F., Thomson L.M., Gillig K.J., Russell D.H. (2002). Observation of conserved solution-phase secondary structure in gas-phase tryptic peptides. J Am Chem Soc.

[102] Bonfiglio R., King R.C., Olah T.V., Merkle K. (1999). The Effects of Sample Preparation Methods on the Variability of the Electrospray Ionization Response for Model Drug Compounds. Rapid Commun Mass Spectrom.

[103] Šedo O., Sedláček I., Zdráhal Z. (2011). Sample preparation methods for MALDI-MS profiling of bacteria. Mass Spectrom Rev.

[104] Nilsson T., Mann M., Aebersold R., Yates J.R., Bairoch A., Bergeron J.J.M. (2010). Mass spectrometry in high-throughput proteomics: ready for the big time. Nat Methods.

[105] Tabb D.L., Vega-Montoto L., Rudnick P.A., Variyath A.M., Ham A.J.L., Bunk D.M. (2009). Repeatability and reproducibility in proteomic identifications by liquid chromatography-tandem mass spectrometry. J Proteome Res.

[106] Haag A.M., Mirzaei H., Carrasco M. (2016). Mass Analyzers and Mass Spectrometers BT - Modern Proteomics – Sample Preparation, Analysis and Practical Applications.

[107] Nordström A., Want E., Northen T., Lehtiö J., Siuzdak G. (2008). Multiple ionization mass spectrometry strategy used to reveal the complexity of metabolomics. Anal Chem.

[108] Bell A.W., Deutsch E.W., Au C.E., Kearney R.E., Beavis R., Sechi S. (2009). A HUPO test sample study reveals common problems in mass spectrometry–based proteomics. Nat Methods.

[109] Boutilier K., Ross M., Podtelejnikov A.V., Orsi C., Taylor R., Taylor P. (2005). Comparison of different search engines using validated MS/MS test datasets. Anal Chim Acta.

[110] Borràs E., Sabidó E. (2017). What is targeted proteomics? A concise revision of targeted acquisition and targeted data analysis in mass spectrometry. Proteomics.

[111] Cole J., Hanson E.J., James D.C., Dockrell D.H., Dickman M.J. (2019). Comparison of data-acquisition methods for the identification and quantification of histone post-translational modifications on a Q Exactive HF hybrid quadrupole Orbitrap mass spectrometer. Rapid Commun Mass Spectrom.

[112] Sinitcyn P., Hamzeiy H., Salinas Soto F., Itzhak D., McCarthy F., Wichmann C. (2021). MaxDIA enables library-based and library-free data-independent acquisition proteomics. Nat Biotechnol.

[113] Ludwig C., Gillet L., Rosenberger G., Amon S., Collins B.C., Aebersold R. (2018). Data-independent acquisition-based SWATH-MS for quantitative proteomics: a tutorial. Mol Syst Biol.

[114] Gillet L.C., Navarro P., Tate S., Röst H., Selevsek N., Reiter L. (2012). Targeted Data Extraction of the MS/MS Spectra Generated by Data-independent Acquisition: A New Concept for Consistent and Accurate Proteome Analysis. Mol Cell Proteom.

[115] Meier F., Geyer P.E., Virreira Winter S., Cox J., Mann M. (2018). BoxCar acquisition method enables single-shot proteomics at a depth of 10,000 proteins in 100 min. Nat Methods.

[116] Meier F., Brunner A.D., Frank M., Ha A., Bludau I., Voytik E. (2020). diaPASEF: parallel accumulation–serial fragmentation combined with data-independent acquisition. Nat Methods.

[117] Crowell K.L., Baker E.S., Payne S.H., Ibrahim Y.M., Monroe M.E., Slysz G.W. (2013). Increasing confidence of LC–MS identifications by utilizing ion mobility spectrometry. Int J Mass Spectrom.

[118] Kösters M., Leufken J., Leidel S.A. (2021). SMITER—a python library for the simulation of LC-MS/MS experiments. Genes (Basel).

