# Supplementary material for: Insight on physicochemical properties governing peptide MS1 response in HPLC-ESI-MS/MS: A deep learning approach
Source: Comput Struct Biotechnol J. 2023 Jul 22;21:3715–27. doi: 10.1016/j.csbj.2023.07.027 (PMC10407266; doi:10.1016/j.csbj.2023.07.027)
Supplement: Supplementary file 1 — Supplementary material [file mmc1.docx]

**Supplementary Material for**

**Insight on Physicochemical Properties Governing Peptide MS1 Response in HPLC-ESI-MS/MS: A Deep Learning Approach**

Naim Abdul-Khalek^1^, Reinhard Wimmer^1^, Michael Toft Overgaard^1^, Simon Gregersen Echers^1,*^.

*^1^ Department of Chemistry and Bioscience, Aalborg University, Fredrik Bajers Vej 7H, Aalborg 9220, Denmark.*

E-mail: sgr@bio.aau.dk (Gregersen Echers, S)

**Running Title:** *Abdul-Khalek N et al / deep learning for Peptide MS Response*

Artificial datasets were generated to build proof-of-concept models for the deep neural network model architecture, namely the encoder-decoder with attention mechanism, to explore its efficacy, capacity, and limitations. Each dataset contained 100.000 training sequences from which 20% were randomly selected as validation dataset. An additional 10.000 sequences were generated as test dataset.

The artificial datasets consist of sequences of numbers instead of amino acids (AAs), where each sequence had a scalar value assigned, emulating an experimental “intensity output value”. Fixed contributions were assigned to each unique numeric element (that would emulate a contributing of each particular AA) of the input sequences and different sets of contributions as well as sequence formats were analyzed for the proof-of-concept models. A simpler example it is shown in Table S1, were there are only 5 possible unique elements that can constituted a sequence, namely the letters A, B, C, D, E. And each unique elements have a fixed contribution assigned, for which each time that a particular element appears in a sequence that contribution will be added to the final “intensity output”. For example, a sequence AACEBD will have an “intensity output” of 87 obtained by adding the contribution of each element 2x1(A) + 1x10(C) + 1x50(E) + 1x5(B) + 1x20(D) = 87

The first dataset format consisted of sequences with a length of 4 to 8 elements comprised of 9 unique numbers (from 1 to 9). Two sets of contribution values were assigned to each unique elements (Table S2) to test the architecture. One set of contributions consisted in an increasingly linear contributions of each unique elements with values ranging from 10 to 90, obtaining mean absolute percentage error (MAPE) of 0.79% and Pearson correlation coefficient (PCC) of 0.999 between the attention weights and the contribution values with a p-value of <3E-11 (Table S3). The second set of contributions consisted of a linear contribution ranging from 1 to 9 for odd numbers and a non-linear contribution ranging from 10 to 160 for even numbers (Table S2). This was done to increase the dynamic range of contributions as well as the gap of contributions between the unique elements. The second set of contributions resulted in a MAPE of 0.56% and PCC of 0.980 with a p-value of <4E-06 (Table S3). In both cases, the attention mechanism successfully identified the relevancy of individual element (Fig. S1) with excellent correlation between real and predicted values (Fig. S2).

The second artificial dataset format consisted of sequences with a length of 7 to 40 elements comprised by 20 unique numbers (from 1 to 20). Similarly, two sets of contribution values were assigned to each unique element (Table S4) to test the architecture. The second dataset format is meant to better emulate the characteristics of the real data, namely the length of the sequences, the order of magnitude of the MS intensity output, and the number of unique elements (20 AA). One set of contributions consisted of an increasingly linear contribution of each unique element with values ranging from 1.0x10^7^ to 2.0x10^8^, obtaining MAPE of 0.60% and PCC of 0.999 with a p-value of <3E-25 (Table S5). The second set consisted of four groups of elements having different orders of magnitude of contributions ranging from 0 to 2.0x10^9^ with a MAPE of 3.15% and PCC of 0.981 with a p-value of <4E-14 (Table S5). Similarly, to the first dataset format, the attention mechanism successfully identified relevant contributions (Fig. S3) and the models obtained strong correlation between predicted and real values (Fig. S4). For all artificial datasets a log-transformation was applied to the output values, which is a common transformation when dealing with non-linear data representing a large dynamic range of values [44–46]. For proof-of-concept models 3 and 4, log-transformations were found a prerequisite to find a solution for the task, while it did not seem to have an impact on the performance of proof-of-concept models 1 and 2.

In general, the models were able to assign the correct contribution of each of the unique elements in the different sequence formats. The lowest correlation was observed with the most complex sequence (20 unique elements) when the fixed contribution values had a very large dynamic range with a maximum contribution of 2x10^9^ and a minimum of 0 (Proof-of-concept model 4). This particular model was unable to assign the correct average contribution of the unique sequence elements which had a contribution lower than 2x10^6^ (3 orders of magnitude below the maximum contribution), although it was able to group elements by order of magnitude of their contribution (Table S6 and Fig. S3B). Nevertheless, all the models were capable of determining the most relevant contributing elements of the sequences in a satisfactory manner and make highly accurate prediction of the output value, thereby validating applicability of the architecture. Moreover, accurate predictions were obtained in scenarios accounting for a uniform distribution of the contribution by each element, but also scenarios accounting for high contrast between contributions of individual elements.

**Supplementary Tables**

**Table S1**: Example of element contribution values for proof-of-concept models’ data design.

| **Element** | **Value** |
| --- | --- |
| **A** | 1 |
| **B** | 5 |
| **C** | 10 |
| **D** | 20 |
| **E** | 50 |

**Table S2**: Element contribution values and obtained attention weights for proof-of-concept models 1 and 2

|  | **Proof-of-concept** | | **Proof-of-concept** | |
| --- | --- | --- | --- | --- |
|  | **Model 1** | | **Model 2** | |
| **Element** | **Value** | **Attention Weight** | **Value** | **Attention Weight** |
| **1** | 10 | 0,123905 | 1 | 0,081468 |
| **2** | 20 | 0,124513 | 10 | 0,112548 |
| **3** | 30 | 0,124988 | 3 | 0,08754 |
| **4** | 40 | 0,125741 | 40 | 0,196395 |
| **5** | 50 | 0,126275 | 5 | 0,093186 |
| **6** | 60 | 0,126778 | 90 | 0,282256 |
| **7** | 70 | 0,12751 | 7 | 0,101281 |
| **8** | 80 | 0,128112 | 160 | 0,350464 |
| **9** | 90 | 0,128624 | 9 | 0,110186 |

**Table S3**: Proof-of-concept models 1 and 2 results showing correlation of attention weights and contribution values (PCC and associated p-value), performance metric (MAPE), and hyperparameter specifications.

|  | **PCC^a^** | **p-value** | **MAPE^b^ (%)** | **Batch Size** | **Units** | **Epochs** | **Output range of values** |  |
| --- | --- | --- | --- | --- | --- | --- | --- | --- |
| **Proof-of-concept**  **Model 1** | | 0.999 | <3E-11 | 0.79 | 8 | 128 | 5 | 1-12 |
| **Proof-of-concept**  **Model 2** | | 0.980 | <4E-06 | 0.56 | 8 | 64 | 20 | 1-12 |

^a^ Pearson correlation coefficient

^b^ Mean absolute percentage error

**Table S4**: Element contribution values and obtained attention weights for proof-of-concept models 3 and 4

|  | **Proof-of-concept** | | **Proof-of-concept** | |
| --- | --- | --- | --- | --- |
|  | **Model 3** | | **Model 4** | |
| **Element** | **Value** | **Attention Weight** | **Value** | **Attention Weight** |
| **1** | 1,0x10^7^ | 0,02216 | 0 | 0,004297 |
| **2** | 2,0x10^7^ | 0,024845 | 6,0x10^6^ | 0,009937 |
| **3** | 3,0x10^7^ | 0,0262 | 3 | 0,003927 |
| **4** | 4,0x10^7^ | 0,029228 | 1,2x10^7^ | 0,014019 |
| **5** | 5,0x10^7^ | 0,031261 | 10 | 0,004406 |
| **6** | 6,0x10^7^ | 0,033604 | 1,8x10^7^ | 0,017216 |
| **7** | 7,0x10^7^ | 0,03581 | 21 | 0,004617 |
| **8** | 8,0x10^7^ | 0,039363 | 2,4x10^7^ | 0,01984 |
| **9** | 9,0x10^7^ | 0,041557 | 36 | 0,004456 |
| **10** | 1,0x 10^8^ | 0,043841 | 3,0x10^7^ | 0,022459 |
| **11** | 1,1x10^8^ | 0,045409 | 1,1x10^6^ | 0,005686 |
| **12** | 1,2x10^8^ | 0,048081 | 7,2x10^8^ | 0,124603 |
| **13** | 1,3x10^8^ | 0,051647 | 1,3x10^6^ | 0,005725 |
| **14** | 1,4x10^8^ | 0,052853 | 8,4x10^8^ | 0,132533 |
| **15** | 1,5x10^8^ | 0,056963 | 1,5x10^6^ | 0,005524 |
| **16** | 1,6x10^8^ | 0,060133 | 1,28x10^9^ | 0,164589 |
| **17** | 1,7x10^8^ | 0,06256 | 1,7x10^6^ | 0,006098 |
| **18** | 1,8x10^8^ | 0,064004 | 1,44x10^9^ | 0,174379 |
| **19** | 1,9x10^8^ | 0,06645 | 1,9x10^6^ | 0,006145 |
| **20** | 2,0x10^8^ | 0,067933 | 2,0x10^9^ | 0,203727 |

**Table S5:** Proof-of-concept models 3 and 4 results showing correlation of attention weights and contribution values (PCC and associated p-value), performance metric (MAPE), and hyperparameter specifications

|  | |  | |  |  |  |  |  |  |
| --- | --- | --- | --- | --- | --- | --- | --- | --- | --- |
|  | **PCC^a^** | **p-value** | | **MAPE^b^ (%)** | **Batch Size** | **Units** | **Epochs** | **Output range of values** |  |
| **Proof-of-concept**  **Model 3** | | 0.999 | <3E-25 | | 0.60 | 32 | 128 | 24 | 1-12 |
| **Proof-of-concept**  **Model 4** | | 0.981 | <4E-14 | | 3.15 | 32 | 256 | 30 | 1-12 |

^a^ Pearson correlation coefficient

^b^ Mean absolute percentage error

**Table S6:** Attention weights of individual amino acids (in descending order) for representative model 1 (Bulky hydrophobic and aromatic amino acids)

| **Amino Acid** | **Attention Weight** |
| --- | --- |
| W | 0,158803 |
| L | 0,140030 |
| F | 0,126985 |
| I | 0,102967 |
| Y | 0,096798 |
| V | 0,083330 |
| P | 0,068007 |
| M | 0,059155 |
| C | 0,035384 |
| R | 0,032246 |
| A | 0,025734 |
| D | 0,021801 |
| N | 0,021531 |
| E | 0,021508 |
| K | 0,021058 |
| H | 0,020838 |
| T | 0,019911 |
| S | 0,019147 |
| Q | 0,018424 |
| G | 0,017545 |

**Table S7**: Relevant properties identified by computing the correlation between attention weights of representative model 1 (Bulky hydrophobic and aromatic amino acids) and the AAindex1

| **Accession number** | **Data description** | **Correlation Score** | **p-value** |
| --- | --- | --- | --- |
| MEEJ810102 | Retention coefficient in NaH2PO4 (Meek-Rossetti, 1981) | 0,94 | 4,1E-10 |
| MEEJ810101 | Retention coefficient in NaClO4 (Meek-Rossetti, 1981) | 0,94 | 8,3E-10 |
| BULH740101 | Transfer free energy to surface (Bull-Breese, 1974) | -0,93 | 1,7E-09 |
| GUOD860101 | Retention coefficient at pH 2 (Guo et al., 1986) | 0,93 | 3,0E-09 |
| PARJ860101 | HPLC parameter (Parker et al., 1986) | -0,93 | 4,1E-09 |
| NOZY710101 | Transfer energy, organic solvent/water (Nozaki-Tanford, 1971) | 0,92 | 8,1E-09 |
| WOLS870101 | Principal property value z1 (Wold et al., 1987) | -0,92 | 9,0E-09 |
| MEEJ800102 | Retention coefficient in HPLC, pH2.1 (Meek, 1980) | 0,92 | 1,5E-08 |
| ZHOH040101 | The stability scale from the knowledge-based atom-atom potential (Zhou-Zhou, | 0,92 | 1,5E-08 |
| VENT840101 | Bitterness (Venanzi, 1984) | 0,91 | 3,8E-08 |
| TAKK010101 | Side-chain contribution to protein stability (kJ/mol) (Takano-Yutani, 2001) | 0,89 | 1,1E-07 |
| ZHOH040102 | The relative stability scale extracted from mutation experiments (Zhou-Zhou, | 0,89 | 1,7E-07 |
| PLIV810101 | Partition coefficient (Pliska et al., 1981) | 0,89 | 1,9E-07 |
| GOLD730101 | Hydrophobicity factor (Goldsack-Chalifoux, 1973) | 0,88 | 2,2E-07 |
| SIMZ760101 | Transfer free energy (Simon, 1976), Cited by Charton-Charton (1982) | 0,88 | 2,8E-07 |
| ARGP820101 | Hydrophobicity index (Argos et al., 1982) | 0,88 | 3,2E-07 |
| JOND750101 | Hydrophobicity (Jones, 1975) | 0,88 | 3,3E-07 |
| ROSM880104 | Hydropathies of amino acid side chains, neutral form (Roseman, 1988) | 0,88 | 1,7E-06 |
| BROC820101 | Retention coefficient in TFA (Browne et al., 1982) | 0,87 | 5,4E-07 |
| CIDH920102 | Normalized hydrophobicity scales for beta-proteins (Cid et al., 1992) | 0,87 | 8,4E-07 |
| ZIMJ680105 | RF rank (Zimmerman et al., 1968) | 0,86 | 1,1E-06 |
| CIDH920105 | Normalized average hydrophobicity scales (Cid et al., 1992) | 0,85 | 1,8E-06 |
| BASU050102 | Interactivity scale obtained by maximizing the mean of correlation | 0,85 | 2,6E-06 |
| MIYS990101 | Relative partition energies derived by the Bethe approximation | -0,84 | 2,9E-06 |
| ZHOH040103 | Buriability (Zhou-Zhou, 2004) | 0,84 | 3,2E-06 |
| MIYS990102 | Optimized relative partition energies - method A (Miyazawa-Jernigan, 1999) | -0,84 | 3,3E-06 |
| WILM950101 | Hydrophobicity coefficient in RP-HPLC, C18 with 0.1%TFA/MeCN/H2O (Wilce et | 0,84 | 3,4E-06 |
| RADA880102 | Transfer free energy from oct to wat (Radzicka-Wolfenden, 1988) | 0,84 | 3,8E-06 |
| FAUJ830101 | Hydrophobic parameter pi (Fauchere-Pliska, 1983) | 0,83 | 4,9E-06 |
| LEVM760106 | van der Waals parameter R0 (Levitt, 1976) | 0,83 | 5,3E-06 |
| WEBA780101 | RF value in high salt chromatography (Weber-Lacey, 1978) | -0,83 | 7,2E-06 |
| BROC820102 | Retention coefficient in HFBA (Browne et al., 1982) | 0,82 | 8,8E-06 |
| CORJ870102 | SWEIG index (Cornette et al., 1987) | 0,81 | 1,2E-05 |
| SWER830101 | Optimal matching hydrophobicity (Sweet-Eisenberg, 1983) | 0,81 | 1,3E-05 |
| BLAS910101 | Scaled side chain hydrophobicity values (Black-Mould, 1991) | 0,81 | 1,4E-05 |
| GUYH850103 | Apparent partition energies calculated from Robson-Osguthorpe index (Guy, | -0,81 | 2,5E-05 |
| LEVM760107 | van der Waals parameter epsilon (Levitt, 1976) | 0,81 | 1,6E-05 |
| ROBB790101 | Hydration free energy (Robson-Osguthorpe, 1979) | 0,81 | 1,7E-05 |
| CIDH920104 | Normalized hydrophobicity scales for alpha/beta-proteins (Cid et al., 1992) | 0,81 | 1,8E-05 |
| WILM950102 | Hydrophobicity coefficient in RP-HPLC, C8 with 0.1%TFA/MeCN/H2O (Wilce et al. | 0,81 | 1,8E-05 |
| MIYS850101 | Effective partition energy (Miyazawa-Jernigan, 1985) | 0,80 | 2,3E-05 |
| ZIMJ680102 | Bulkiness (Zimmerman et al., 1968) | 0,80 | 2,4E-05 |
| CIDH920103 | Normalized hydrophobicity scales for alpha+beta-proteins (Cid et al., 1992) | 0,80 | 2,4E-05 |
| BASU050101 | Interactivity scale obtained from the contact matrix (Bastolla et al., 2005) | 0,80 | 2,5E-05 |
| OOBM770103 | Long range non-bonded energy per atom (Oobatake-Ooi, 1977) | -0,80 | 2,8E-05 |
| EISD860101 | Solvation free energy (Eisenberg-McLachlan, 1986) | 0,79 | 3,3E-05 |
| MEEJ800101 | Retention coefficient in HPLC, pH7.4 (Meek, 1980) | 0,79 | 4,0E-05 |
| GRAR740102 | Polarity (Grantham, 1974) | -0,78 | 5,3E-05 |
| COWR900101 | Hydrophobicity index, 3.0 pH (Cowan-Whittaker, 1990) | 0,78 | 5,7E-05 |
| NISK860101 | 14 A contact number (Nishikawa-Ooi, 1986) | 0,78 | 5,8E-05 |
| ROSG850101 | Mean area buried on transfer (Rose et al., 1985) | 0,77 | 6,3E-05 |
| ROSM880105 | Hydropathies of amino acid side chains, pi-values in pH 7.0 (Roseman, 1988) | 0,77 | 1,1E-04 |
| BASU050103 | Interactivity scale obtained by maximizing the mean of correlation | 0,77 | 7,1E-05 |
| GARJ730101 | Partition coefficient (Garel et al., 1973) | 0,77 | 7,5E-05 |
| PONP800107 | Accessibility reduction ratio (Ponnuswamy et al., 1980) | 0,77 | 7,7E-05 |
| CIDH920101 | Normalized hydrophobicity scales for alpha-proteins (Cid et al., 1992) | 0,77 | 8,4E-05 |
| KIDA850101 | Hydrophobicity-related index (Kidera et al., 1985) | -0,76 | 9,3E-05 |
| WIMW960101 | Free energies of transfer of AcWl-X-LL peptides from bilayer interface to | 0,76 | 9,6E-05 |
| MIYS990105 | Optimized relative partition energies - method D (Miyazawa-Jernigan, 1999) | -0,76 | 1,0E-04 |
| MEIH800101 | Average reduced distance for C-alpha (Meirovitch et al., 1980) | -0,76 | 1,1E-04 |
| VINM940102 | Normalized flexibility parameters (B-values) for each residue surrounded by | -0,76 | 1,1E-04 |
| MIYS990104 | Optimized relative partition energies - method C (Miyazawa-Jernigan, 1999) | -0,76 | 1,1E-04 |
| BIOV880101 | Information value for accessibility; average fraction 35% (Biou et al., 1988) | 0,75 | 1,2E-04 |
| LEVM760101 | Hydrophobic parameter (Levitt, 1976) | -0,75 | 1,4E-04 |
| BULH740102 | Apparent partial specific volume (Bull-Breese, 1974) | 0,75 | 1,5E-04 |
| OOBM770104 | Average non-bonded energy per residue (Oobatake-Ooi, 1977) | -0,75 | 1,6E-04 |
| BIOV880102 | Information value for accessibility; average fraction 23% (Biou et al., 1988) | 0,74 | 1,9E-04 |
| WERD780101 | Propensity to be buried inside (Wertz-Scheraga, 1978) | 0,73 | 2,3E-04 |
| HOPT810101 | Hydrophilicity value (Hopp-Woods, 1981) | -0,73 | 2,4E-04 |
| LAWE840101 | Transfer free energy, CHP/water (Lawson et al., 1984) | 0,73 | 2,7E-04 |
| NAKH900110 | Normalized composition of membrane proteins (Nakashima et al., 1990) | 0,73 | 2,7E-04 |
| AVBF000109 | Slopes proteins, FDPB VFF neutral (Avbelj, 2000) | 0,73 | 4,3E-04 |
| ROSM880102 | Side chain hydropathy, corrected for solvation (Roseman, 1988) | -0,72 | 3,2E-04 |
| KARP850101 | Flexibility parameter for no rigid neighbors (Karplus-Schulz, 1985) | -0,72 | 3,3E-04 |
| GUYH850102 | Apparent partition energies calculated from Wertz-Scheraga index (Guy, 1985) | -0,72 | 3,3E-04 |
| MIYS990103 | Optimized relative partition energies - method B (Miyazawa-Jernigan, 1999) | -0,72 | 3,3E-04 |
| RACS770101 | Average reduced distance for C-alpha (Rackovsky-Scheraga, 1977) | -0,72 | 3,6E-04 |
| RADA880108 | Mean polarity (Radzicka-Wolfenden, 1988) | 0,71 | 4,2E-04 |
| PUNT030102 | Knowledge-based membrane-propensity scale from 3D_Helix in MPtopo databases | -0,71 | 4,5E-04 |
| RICJ880107 | Relative preference value at N4 (Richardson-Richardson, 1988) | 0,71 | 4,9E-04 |
| PARS000101 | p-Values of mesophilic proteins based on the distributions of B values | -0,71 | 5,0E-04 |
| ROSM880101 | Side chain hydropathy, uncorrected for solvation (Roseman, 1988) | -0,70 | 5,3E-04 |

**Table S8**: Attention weights of individual amino acids (in descending order) for representative model 2 (Positively charged amino acids)

| **Amino Acids** | **Attention Weights** |
| --- | --- |
| R | 0,378370 |
| K | 0,366475 |
| H | 0,172637 |
| P | 0,044171 |
| L | 0,043256 |
| G | 0,042278 |
| S | 0,038368 |
| A | 0,037726 |
| I | 0,037630 |
| N | 0,032570 |
| W | 0,032282 |
| V | 0,031488 |
| F | 0,029724 |
| C | 0,029234 |
| Q | 0,029042 |
| M | 0,026585 |
| D | 0,026067 |
| E | 0,025886 |
| Y | 0,023940 |
| T | 0,023057 |

**Table S9**: Relevant properties identified by computing the correlation between attention weights of representative model 2 (Positively charged amino acids) and the AAindex1

| **Accession number** | **Data description** | **Correlation Score** | **p-value** |
| --- | --- | --- | --- |
| FAUJ880111 | Positive charge (Fauchere et al., 1988) | 0,93 | 1,6E-09 |
| ZIMJ680104 | Isoelectric point (Zimmerman et al., 1968) | 0,88 | 4,4E-07 |
| EISD860102 | Atom-based hydrophobic moment (Eisenberg-McLachlan, 1986) | 0,84 | 3,5E-06 |
| FINA910103 | Helix termination parameter at posision j-2,j-1,j (Finkelstein et al., 1991) | 0,78 | 4,9E-05 |
| HUTJ700103 | Entropy of formation (Hutchens, 1970) | 0,77 | 7,6E-05 |
| JACR890101 | Weights from the IFH scale (Jacobs-White, 1989) | -0,76 | 1,0E-04 |
| RADA880107 | Energy transfer from out to in(95%buried) (Radzicka-Wolfenden, 1988) | -0,75 | 1,6E-04 |
| KLEP840101 | Net charge (Klein et al., 1984) | 0,74 | 1,8E-04 |
| JANJ780101 | Average accessible surface area (Janin et al., 1978) | 0,72 | 3,5E-04 |

**Table S10**: Performance metrics (expressed as MAPE (%) on real and log-transformed scale) and hyperparameter specifications for the two representative models and the final model (specific/tryptic data subset)

|  | **MAPE^a^ (%)** | **MAPE^a^ (%)**  **Log-Transformed** | **Batch Size** | **Units** | **Epochs** | **Output range of values** |
| --- | --- | --- | --- | --- | --- | --- |
| **Representative Model 1** | 188.3 ± 8.3 | 12.61 ± 0.41 | 128 | 128 | 5 | 1-5 |
| **Representative Model 2** | 250.1 ± 6.9 | 16.94 ± 0.36 | 512 | 256 | 5 | 1-24 |
| **Final Model** | 97.5 ± 6.2 | 9.67 ± 0.53 | 256 | 128 | 10 | 1-5 |

^a^ Mean absolute percentage error

**Table S11:** Attention weights of individual amino acids (descending weight) for the final model

| **Amino Acid** | **Attention Weight** |
| --- | --- |
| W | 0,151849 |
| F | 0,119770 |
| L | 0,112485 |
| Y | 0,099900 |
| I | 0,094913 |
| P | 0,088325 |
| V | 0,084304 |
| N | 0,059511 |
| M | 0,056819 |
| T | 0,052511 |
| E | 0,046911 |
| A | 0,046023 |
| R | 0,045235 |
| K | 0,045145 |
| Q | 0,044501 |
| C | 0,043824 |
| S | 0,041541 |
| D | 0,039935 |
| H | 0,039882 |
| G | 0,031428 |

**Table S12:** Relevant properties identified by computing the correlation between attention weights of the final model and AAindex1

| **Accession number** | **Data description** | **Correlation Score** | **p-value** |
| --- | --- | --- | --- |
| MEEJ800102 | Retention coefficient in HPLC, pH2.1 (Meek, 1980) | 0,91 | 2,6E-08 |
| MEEJ810102 | Retention coefficient in NaH2PO4 (Meek-Rossetti, 1981) | 0,90 | 4,3E-08 |
| NOZY710101 | Transfer energy, organic solvent/water (Nozaki-Tanford, 1971) | 0,90 | 6,2E-08 |
| TAKK010101 | Side-chain contribution to protein stability (kJ/mol) (Takano-Yutani, 2001) | 0,90 | 8,7E-08 |
| GOLD730101 | Hydrophobicity factor (Goldsack-Chalifoux, 1973) | 0,89 | 1,3E-07 |
| MEEJ810101 | Retention coefficient in NaClO4 (Meek-Rossetti, 1981) | 0,88 | 2,3E-07 |
| BULH740101 | Transfer free energy to surface (Bull-Breese, 1974) | -0,88 | 4,4E-07 |
| ZHOH040101 | The stability scale from the knowledge-based atom-atom potential (Zhou-Zhou, | 0,87 | 4,6E-07 |
| ZHOH040102 | The relative stability scale extracted from mutation experiments (Zhou-Zhou, | 0,87 | 5,8E-07 |
| VENT840101 | Bitterness (Venanzi, 1984) | 0,87 | 6,5E-07 |
| WOLS870101 | Principal property value z1 (Wold et al., 1987) | -0,87 | 7,3E-07 |
| SIMZ760101 | Transfer free energy (Simon, 1976), Cited by Charton-Charton (1982) | 0,87 | 7,3E-07 |
| GUOD860101 | Retention coefficient at pH 2 (Guo et al., 1986) | 0,87 | 7,9E-07 |
| ARGP820101 | Hydrophobicity index (Argos et al., 1982) | 0,86 | 9,4E-07 |
| JOND750101 | Hydrophobicity (Jones, 1975) | 0,86 | 9,4E-07 |
| WEBA780101 | RF value in high salt chromatography (Weber-Lacey, 1978) | -0,86 | 1,5E-06 |
| PARJ860101 | HPLC parameter (Parker et al., 1986) | -0,85 | 1,9E-06 |
| LEVM760107 | van der Waals parameter epsilon (Levitt, 1976) | 0,85 | 2,0E-06 |
| ZIMJ680105 | RF rank (Zimmerman et al., 1968) | 0,84 | 3,4E-06 |
| BROC820101 | Retention coefficient in TFA (Browne et al., 1982) | 0,84 | 3,9E-06 |
| GARJ730101 | Partition coefficient (Garel et al., 1973) | 0,82 | 1,0E-05 |
| BROC820102 | Retention coefficient in HFBA (Browne et al., 1982) | 0,82 | 1,1E-05 |
| WILM950101 | Hydrophobicity coefficient in RP-HPLC, C18 with 0.1%TFA/MeCN/H2O (Wilce et | 0,81 | 1,3E-05 |
| ZIMJ680102 | Bulkiness (Zimmerman et al., 1968) | 0,81 | 1,8E-05 |
| PLIV810101 | Partition coefficient (Pliska et al., 1981) | 0,81 | 1,8E-05 |
| RADA880102 | Transfer free energy from oct to wat (Radzicka-Wolfenden, 1988) | 0,80 | 2,2E-05 |
| CIDH920102 | Normalized hydrophobicity scales for beta-proteins (Cid et al., 1992) | 0,80 | 2,5E-05 |
| LEVM760106 | van der Waals parameter R0 (Levitt, 1976) | 0,79 | 2,9E-05 |
| ROSM880104 | Hydropathies of amino acid side chains, neutral form (Roseman, 1988) | 0,79 | 8,8E-05 |
| WILM950102 | Hydrophobicity coefficient in RP-HPLC, C8 with 0.1%TFA/MeCN/H2O (Wilce et al. | 0,79 | 3,5E-05 |
| MEEJ800101 | Retention coefficient in HPLC, pH7.4 (Meek, 1980) | 0,79 | 4,0E-05 |
| AVBF000109 | Slopes proteins, FDPB VFF neutral (Avbelj, 2000) | 0,78 | 9,7E-05 |
| CIDH920105 | Normalized average hydrophobicity scales (Cid et al., 1992) | 0,77 | 6,8E-05 |
| ZHOH040103 | Buriability (Zhou-Zhou, 2004) | 0,77 | 6,9E-05 |
| FAUJ830101 | Hydrophobic parameter pi (Fauchere-Pliska, 1983) | 0,77 | 7,0E-05 |
| OOBM770104 | Average non-bonded energy per residue (Oobatake-Ooi, 1977) | -0,77 | 7,3E-05 |
| BASU050102 | Interactivity scale obtained by maximizing the mean of correlation | 0,76 | 9,6E-05 |
| BLAS910101 | Scaled side chain hydrophobicity values (Black-Mould, 1991) | 0,76 | 1,0E-04 |
| EISD860101 | Solvation free energy (Eisenberg-McLachlan, 1986) | 0,75 | 1,2E-04 |
| LEVM760101 | Hydrophobic parameter (Levitt, 1976) | -0,74 | 1,7E-04 |
| WIMW960101 | Free energies of transfer of AcWl-X-LL peptides from bilayer interface to | 0,74 | 2,1E-04 |
| ROSG850101 | Mean area buried on transfer (Rose et al., 1985) | 0,73 | 2,3E-04 |
| CORJ870102 | SWEIG index (Cornette et al., 1987) | 0,73 | 2,6E-04 |
| GUYH850103 | Apparent partition energies calculated from Robson-Osguthorpe index (Guy, | -0,73 | 3,9E-04 |
| SWER830101 | Optimal matching hydrophobicity (Sweet-Eisenberg, 1983) | 0,73 | 2,7E-04 |
| MIYS990101 | Relative partition energies derived by the Bethe approximation | -0,73 | 2,8E-04 |
| MIYS990102 | Optimized relative partition energies - method A (Miyazawa-Jernigan, 1999) | -0,73 | 3,0E-04 |
| ROSM880105 | Hydropathies of amino acid side chains, pi-values in pH 7.0 (Roseman, 1988) | 0,72 | 4,9E-04 |
| ROBB790101 | Hydration free energy (Robson-Osguthorpe, 1979) | 0,72 | 3,3E-04 |
| CIDH920104 | Normalized hydrophobicity scales for alpha/beta-proteins (Cid et al., 1992) | 0,72 | 3,5E-04 |
| HOPT810101 | Hydrophilicity value (Hopp-Woods, 1981) | -0,71 | 4,1E-04 |
| OOBM770103 | Long range non-bonded energy per atom (Oobatake-Ooi, 1977) | -0,71 | 4,4E-04 |
| COWR900101 | Hydrophobicity index, 3.0 pH (Cowan-Whittaker, 1990) | 0,71 | 4,7E-04 |
| KIDA850101 | Hydrophobicity-related index (Kidera et al., 1985) | -0,71 | 5,2E-04 |
| BASU050101 | Interactivity scale obtained from the contact matrix (Bastolla et al., 2005) | 0,70 | 5,5E-04 |
| LAWE840101 | Transfer free energy, CHP/water (Lawson et al., 1984) | 0,70 | 5,8E-04 |

**Supplementary Figures**


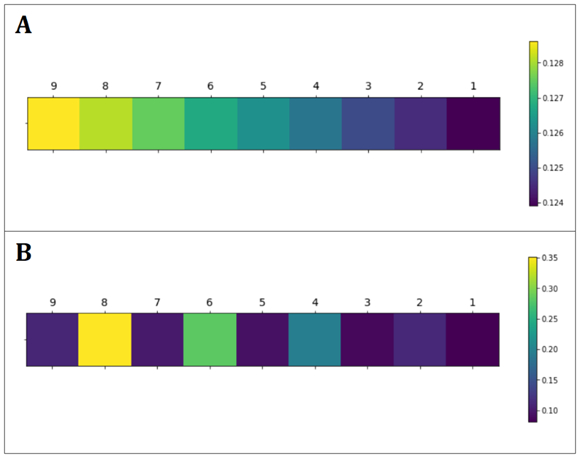


**Figure S1:** **Graphical representation of obtained attention weights for proof-of-concept models 1 (A) and 2 (B).** The color coding indicates the assigned contribution of each AA to the prediction of the MS1 intensity output from high contribution (yellow) gradually decreasing to low contribution (dark blue).


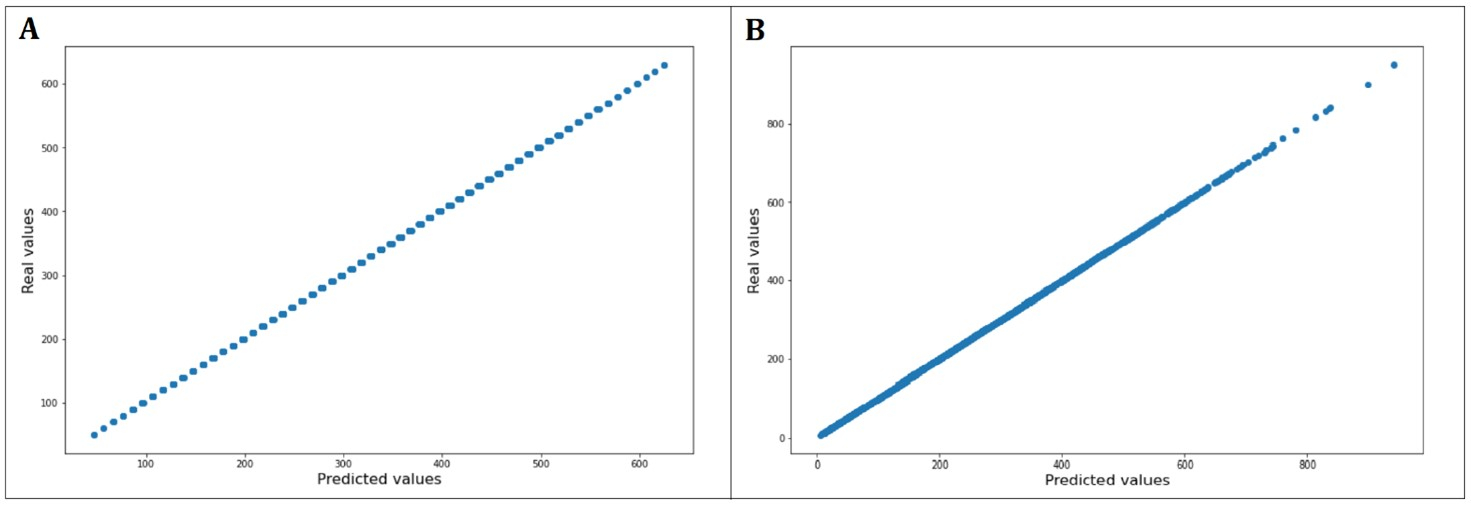


**Figure S2**: **Graphical representation of the real versus predicted output values for proof-of-concept models 1 (A) and 2 (B).**

**
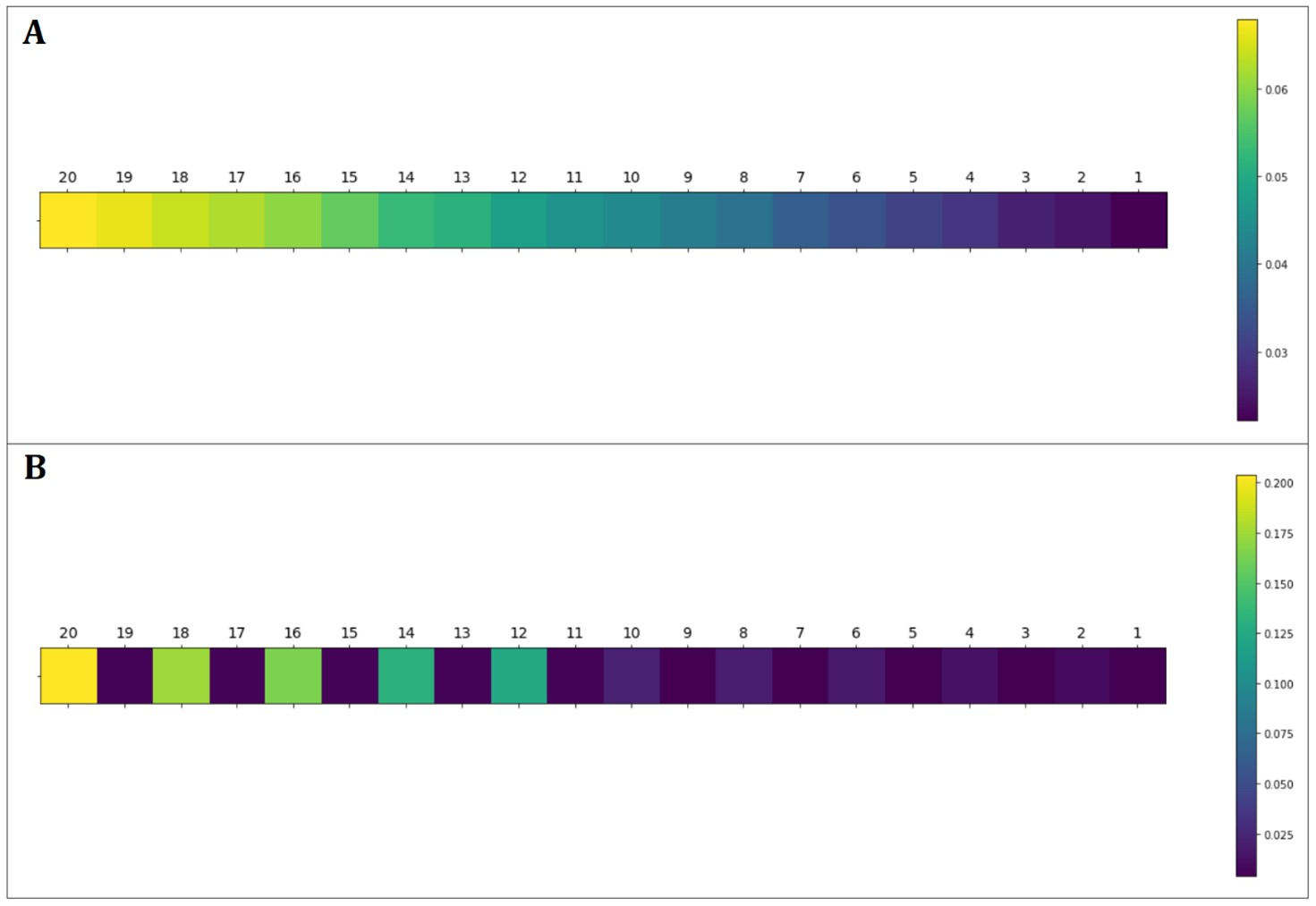
**

**Figure S3**: **Graphical representation of attention weights for proof-of-concept models 3 (A) and 4 (B).** The color coding indicates the assigned contribution of each AA to the prediction of the MS1 intensity output from high contribution (yellow) gradually decreasing to low contribution (dark blue).

**
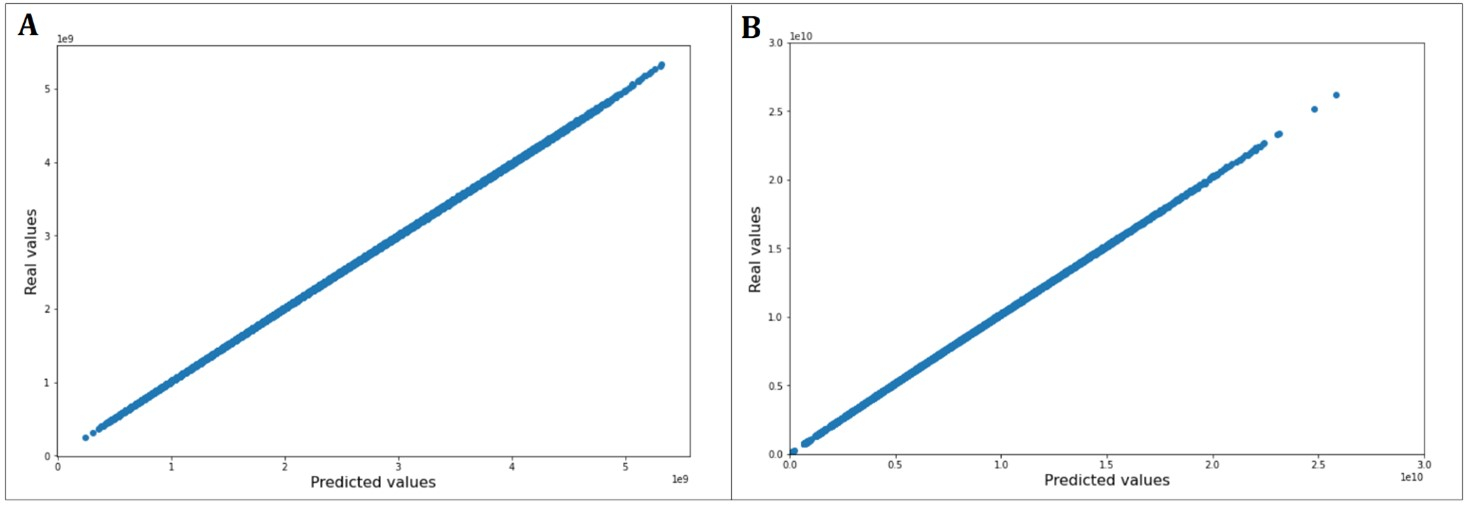
**

**Figure S4**: **Graphical representation of the real versus predicted output values for proof-of-concept models 3 (A) and 4 (B).**


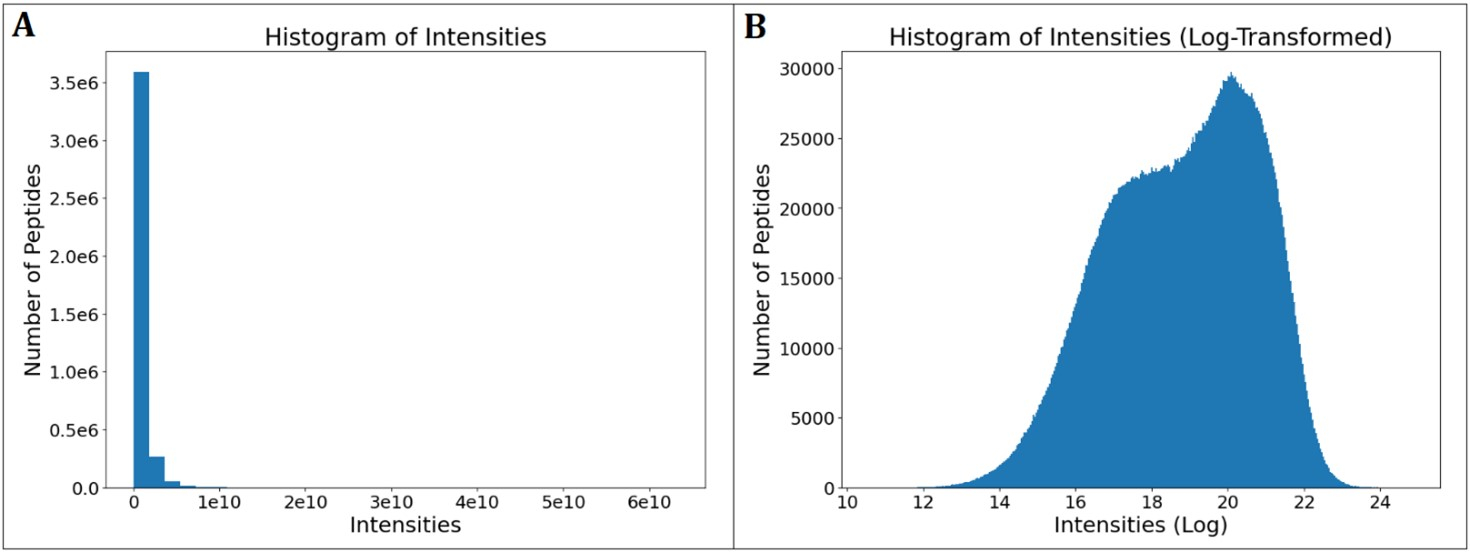


**Figure S5**: **Distribution of the unfiltered data. A.** Histogram of intensity distributions showing raw MS1 intensities (Bin size ≈ 1.8e9). **B.** Histogram of log-transformed MS1 intensities (Bin size ≈ 0.038).


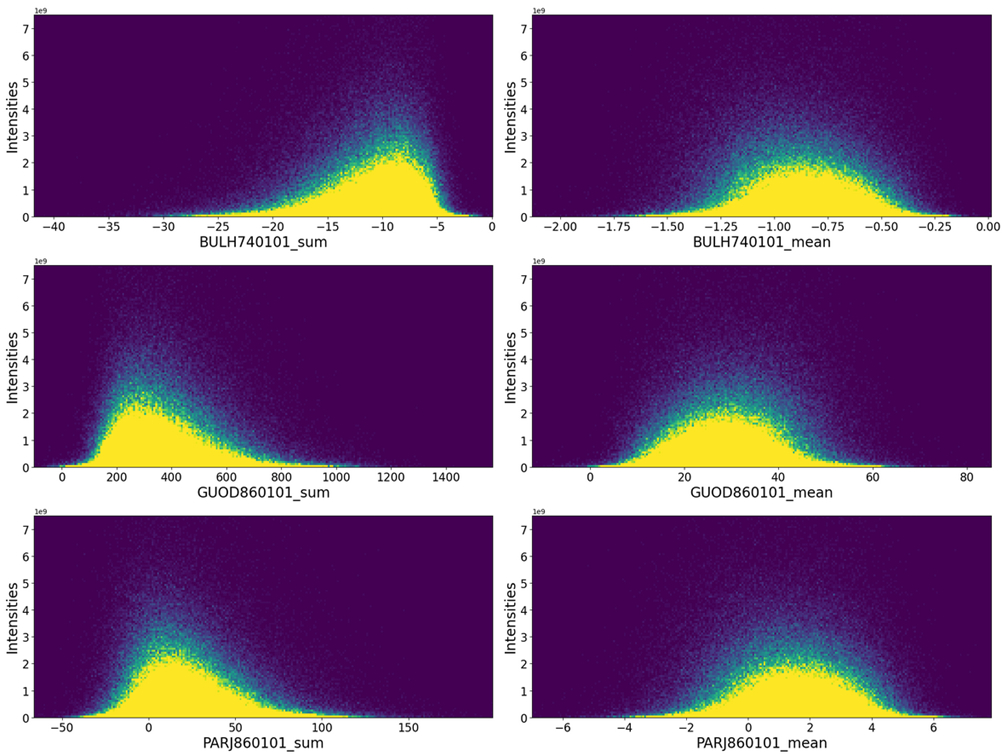


**Figure S6:** **2D histograms of MS1 intensities versus sum (left) and mean (right) computed hydrophobic properties.** Hydrophobic properties of peptides are based on AAindex indices BULH740101 (top), GUOD860101 (middle), and PARJ860101 (bottom) for the filtered dataset. The color coding indicates point density going from 0 (dark blue) to 40 (yellow). There are 250 bins in each axis, and the Y axis has been set a maximum intensity value of 7.5e9.


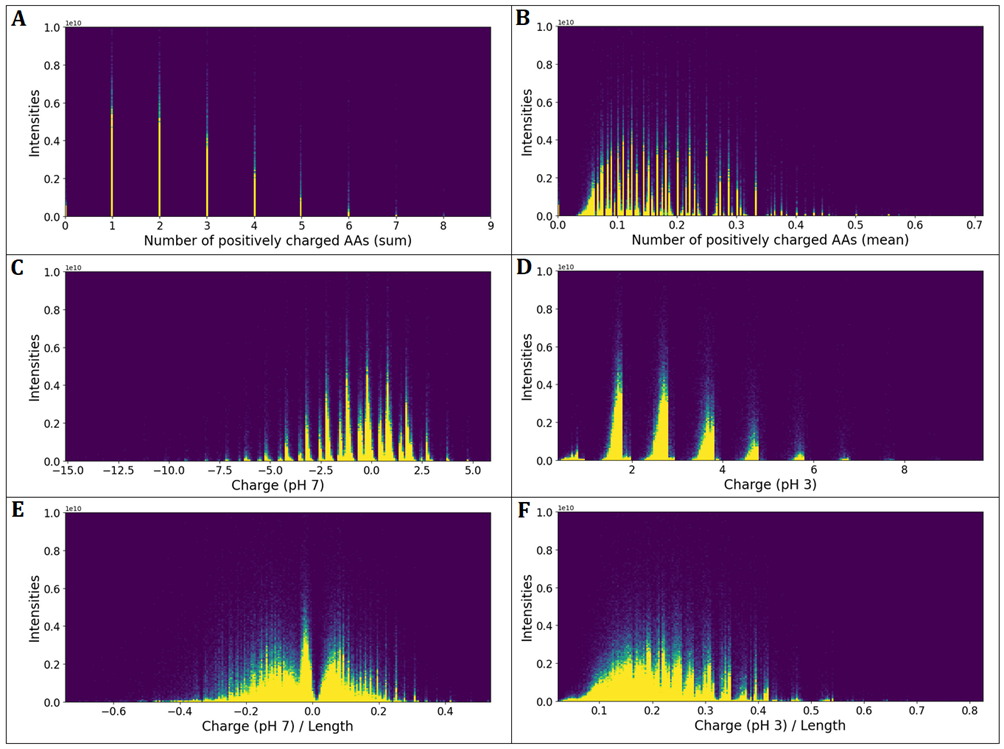


**Figure S7:** **2D histograms of MS1 intensities versus of computed charge for the filtered dataset.** **A.** Sum of positively charged AAs. **B.** Mean of positively charged AAs. **C.** Charge at pH 7. **D.** Charge at pH 3. **E.** Charge at pH 7 (normalized by peptide length). **F.** Charge at pH 3 (normalized by peptide length). The color coding indicates point density going from 0 (dark blue) to 40 (yellow). There are 250 bins in each axis, and the Y axis has been set a maximum intensity value of 1.0e10.

**
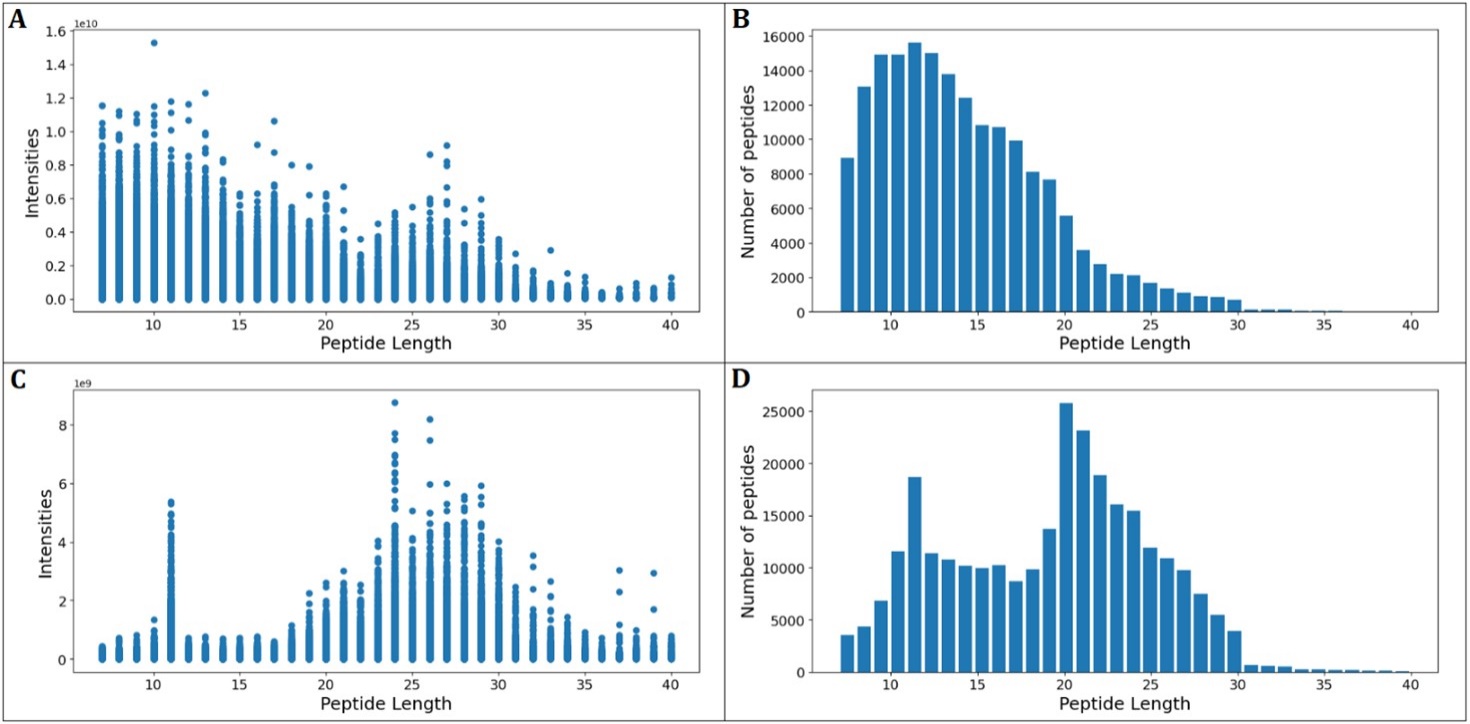
**

**Figure S8**: **Graphical Representation of peptides for the filtered dataset and peptides identified with MaxQuant semi-specific search mode.** **A.** Scatter plot of peptide length versus intensity in filtered dataset. **B.** Histogram of peptides length in filtered dataset. **C.** Scatter plot of peptide length versus intensity with MaxQuant semi-specific setting. **D.** Histogram of peptides length with MaxQuant semi-specific setting.

**
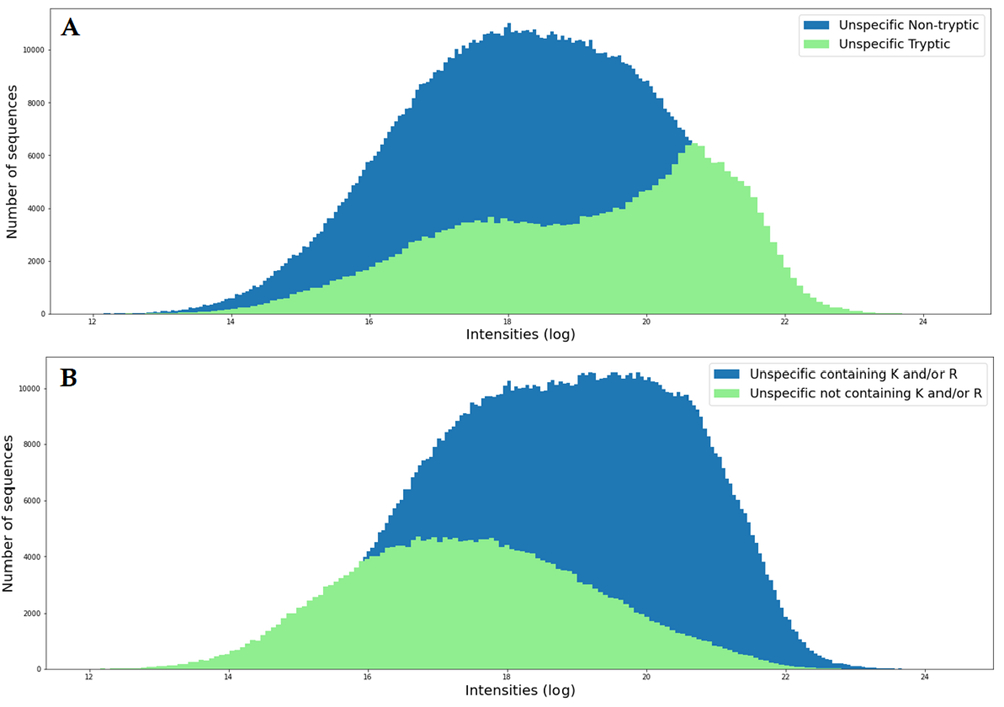
**

**Figure S9**: **Histograms of peptides (Unspecific) and their log transform intensities.** **A.** Histogram with all tryptic and non- tryptic peptides identified using unspecific search mode. **B.** Histogram of all peptides with and without K and/or R anywhere in the sequence, identified using unspecific search mode.


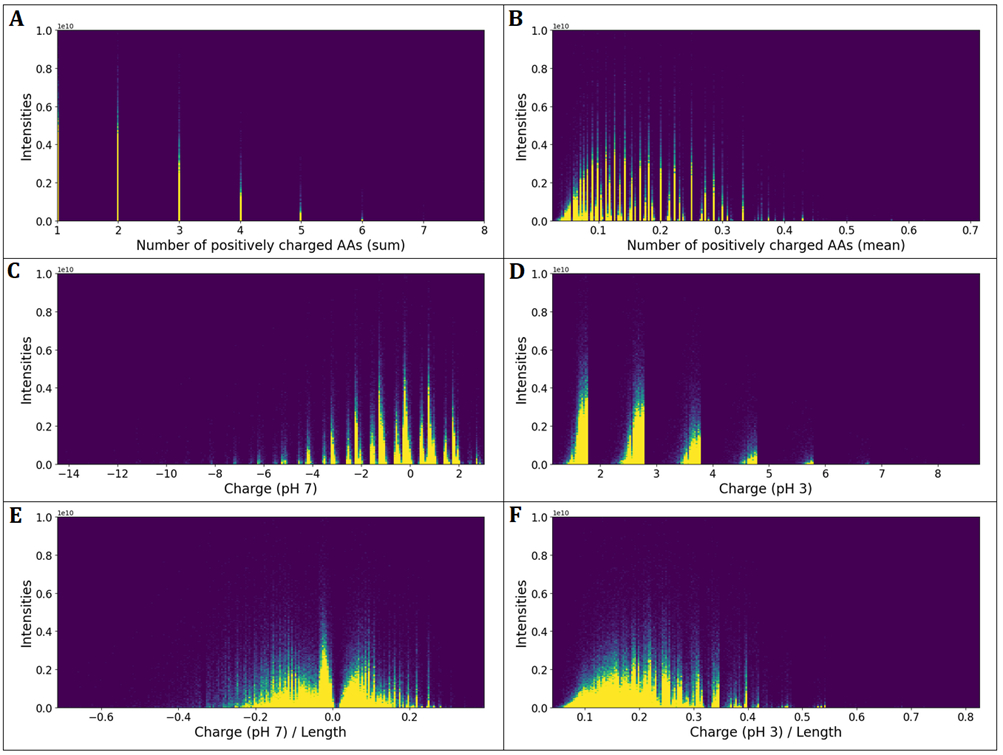


**Figure S10:** **2D histograms of MS1 intensities versus of computed charge for individual peptides in the specific/tryptic data subset.** **A.** Sum of positively charged AAs. **B.** Mean of positively charged AAs. **C.** Charge at pH 7. **D.** Charge at pH 3. **E.** Charge at pH 7 over peptide length. **F.** Charge at pH 3 over peptide length. The color coding indicates point density going from 0 (dark blue) to 40 (yellow). There are 250 bins in each axis, and the Y axis has been set a maximum intensity value of 1.0e10.


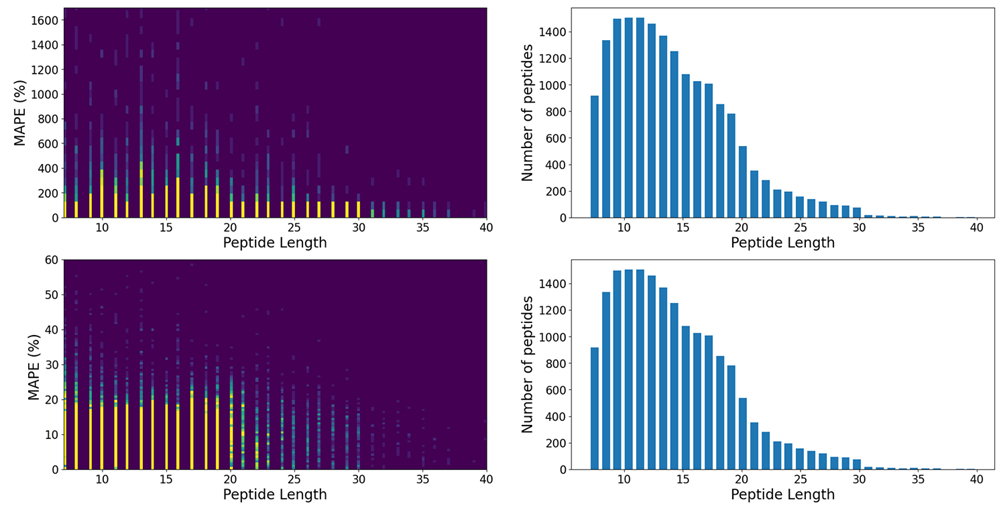


**Figure S11:** **Distribution of MAPE for predicted MS1 intensities as a function of peptide length for the specific/tryptic test dataset.** Left: 2D histograms of MAPE for predicted MS1 intensities prediction versus peptide length in real scale (top) and log-transformed (bottom). The color coding indicates point density going from 0 (dark blue) to 15 (yellow). There are 150 bins in each axis, and the Y axis has been set a maximum intensity value of 1700 (top) and 60 (bottom). Right: Histograms of peptide length (top and bottom are identical).

**END-TO-END PIPELINE WITH LIBRARIES, VERSIONS, AND SETTINGS**

1. **Data extraction**

Initially, the MaxQuant result zip files corresponding to all the 1000 peptides pools were retrieved from their repositories (PXD004732 [38], PXD010595 [39], and PXD021013 [40]), as well as their Sample and Data Relationship Files (SDRF). Within each zip file (corresponding to each pool), there are contained two particular files of interest: peptides.txt and summary.txt. A CSV file was generated containing the information of interest of each pool and the SDRF file by using a custom Python script:

- From the peptides.txt file all the information was extracted.
- From the summary.txt file the enzyme and enzyme mode setting used in the MaxQuant analysis when processing the raw MS output was also obtained.
- The information from the corresponding SDRF file was also included in the CSV file for each pool.

Finally, all CSV files were combined into a single CSV containing the information mentioned previously for all the pools from all the repositories.

1. **Data processing**

An initial filtering was done to secure the quality of the data:

- Only peptides with a PEP score lesser than 0.01 were kept.
- Peptides that were identified with the reverse sequence were removed.
- Peptides identified as contaminants were also excluded.
- Peptides with an intensity value of 0 were discarded.

**Note:** PEP score, reverse sequence and Potential contaminant information were obtained from the peptides.txt files.

After the initial filtering the data was further filtered in two different ways. One that was used to build the representative models 1 and 2, and another one to build the final model.

Data filtering 1 (for representative models):

- Replicated peptide sequences were removed keeping the median intensity value.
- The coefficient of variation (CV) of the intensity for repeated sequences was calculated. Keeping those peptides with equal or lower than 0.3. Thus, none repeated peptides were also eliminated from the database.

Data filtering 2 (for final models):

- Only peptides that were processed Specific enzyme setting with MaxQuant were kept.
- Only tryptic peptides were considered.
- The same filtering done in data filtering 1 was applied.

**Note:** Enzyme and enzyme mode were obtained from the summary.txt files.

Further data processing:

- The peptide sequences were one-hot-encoded. The peptide sequences are the input values for the models.
- The data was split for doing a 5-fold cross-validation.
- MS1 intensity values were log transformed (natural logarithm).
- The log transformed intensities were scaled to a specific range of values. The results from this final transformation are the output values for the model or the dependent variable. The transformation was fit on the training data and applied to the training and test data. This was done for the dataset used to build the representative models and the final model.
- The training data was further split into training data (80%) and validation data (20%).
- All the one-hot-encoded peptide sequences were padded to a maximum length of 40 amino acids.

1. **Model Architecture**

The models’ architecture (encoder-decoder with attention) was build using Tensorflow/Keras subclassing API and with customized training and validation loops.

- 1. **Workflow:**

1. Generate input sequences X = {x_1_, x_2,_ x_3, …,_ x_T_} and corresponding output values Y= {y_1_, y_2,_ y_3, …,_ y_T_}.
2. Split the data (5-fold cross-validation (train, validation, and test), batches)
3. For each fold from 1 to 5 DO:
4. For each epoch DO:
5. For each batch size DO:
6. For each time step j from 1 to T DO: #Encoder
7. *h_j_* = BiGRU(*x_j_*, *h_j-1_*) -> *h* = {*h_1_*, *h_2_*_,_ *h_3_*_, …,_ *h_T_*}, where *h* is all hidden state of the encoder.
8. *s_0_* = *h_T_*, where *s_0_* is the first initial hidden state of the decoder. If there were more than one time step the decoder initial hidden state for *i* would be (*s_i-1_*).
9. For time step *i* from 1 to 1 DO (only one time step since the output it just a scalar value): #Decoder
10. *e_ij_* = *v* tanh(*Ws_i-1_* + *Uh_j_*), where *e_ij_* is the attention scores, *W* and *U* are weight matrices and *v* is a weight vector.
11. *α_ij_* = Softmax(*e_ij_*), where *α_ij_* is the attention weights.
12. $c_{i}= \sum_{j=1}^{T} \alpha_{ij}h_{j}$, where *c_i_* is the context vector.
13. *y_0_* = concat(start, *c_i_*), *y_0_* is the first decoder input, start is a initializing character. If there were more than one time step the start would be replace by the previous encoder output (*y_i-1_*).
14. *s_i_* = BiGRU(*y_i-1_*, *s_i-1_*)
15. *y_i_* = Dense(*s_i_*)
16. Compute loss
17. Compute gradient and optimize weights (Adam)
18. Return trained model.
    1. **Libraries, functions and settings:**
       1. **Libraries:**

The main libraries used in this study are:

- TensorFlow (v. 2.5.0) is an end-to-end open-source software library for machine learning and artificial intelligence. Used for: Process data and build machine learning models.
- Scikit-learn (v. 0.24.1) is a machine learning library. Used for: Process data and build models.
- Pandas (v.1.2.4) is an open-source Python library for data analysis and manipulation. Used for: Reading, filtering, and processing the data.
- Matplotlib (v.3.3.4) is library for data visualization. Used for: Generating plots.
- Seaborn (v.0.11.1) is also a library for data visualization based on matplotlib. Use for: Generating plots.
- SciPy (v.1.6.2) is a scientific computation library that uses NumPy. Used for: Different computation (e.g., Pearson correlation).
- NumPy (v.1.20.1) is also a library for scientific computing with Python. Used for: Working with n-dimensional arrays, numerical computing tools.
  - 1. **Functions and settings:**

**Data processing:**

To split the data:

- Function: KFold (sklearn/model_selection). Settings: n_splits = 5, shuffle = True, random_state = an integer value (e.g., 13).
- Function: train_test_split (sklearn/model_selection). Settings: test_size = the test size (e.g., 0.20), random_state = an integer value (e.g., 13).

To transform the intensity values:

- Function: FunctionTransformer (sklearn/preprocessing). Settings: func = numpy.log, inverse_func = numpy.exp, validate = True, check_inverse = True.
- Function: MinMaxScaler (sklearn/preprocessing). Settings: the specific range of values.

To pad the sequences:

- Function: pad_sequences (tensorflow/keras/preprocessing/sequence.). Settings: maxlen = 40, padding = 'post', dtype = 'float32', value = the one-hot-encoded padding (e.g., [1, 0, 0, 0, 0, 0, 0, 0, 0, 0, 0, 0, 0, 0, 0, 0, 0, 0, 0, 0, 0])

To generate the batches:

- from_tensor_slices (tensorflow/data/Dataset)

**Encoder:**

The encoder consists of one bidirectional GRU layer:

Functions from tensorflow/keras/layers:

- GRU. Settings: units: number of cells, return_sequences = True, return_state = True, recurrent_initializer='glorot_uniform'.
- Bidirectional. Settings: layer = GRU layer.
- Masking. Settings: mask_value = one-hot-encoded padding character/value.

The encoder has as input the data, as well as the hidden states and the mask.

**Attention Mechanism:**

Functions from tensorflow/keras/layers:

- Dense. Settings: units = number of cells.

**Decoder:**

The decoder consists of one bidirectional GRU layer and one dense layer:

Functions from tensorflow/keras/layers:

- GRU. Settings: units: number of cells, return_state = True, recurrent_initializer='glorot_uniform'.
- Bidirectional. Settings: layer = GRU layer.
- Dense. Settings: units = number of cells.

The decoder has as input the context vector (from the Attention Mechanism) plus the previous decoder output (start character), and the initial hidden states (last hidden state from the encoder).

**Training loop:**

Functions:

- Adam (tensorflow/keras/optimizers). Settings: default.
- MeanSquaredError (tensorflow/keras/losses)
- MeanAbsoluteError (tensorflow/keras/metrics)
- GradientTape (tensorflow)
